# The influence of PI3K inhibition on the radiotherapy response of head and neck cancer cells

**DOI:** 10.1038/s41598-020-73249-z

**Published:** 2020-10-01

**Authors:** Mary Glorieux, Rüveyda Dok, Sandra Nuyts

**Affiliations:** 1 Laboratory of Experimental Radiotherapy, Department of Oncology, KU Leuven, University of Leuven, 3000 Leuven, Belgium; 2 Department of Radiation Oncology, Leuven Cancer Institute, UZ Leuven, 3000 Leuven, Belgium

**Keywords:** Head and neck cancer, Radiotherapy

## Abstract

Radiotherapy has a central role in the treatment of head and neck squamous cell carcinoma (HNSCC). Activation of the PI3K/AKT/mTOR pathway can decrease the efficiency of radiotherapy via the promotion of cell survival and DNA repair. Here, the influence of PI3K pathway inhibition on radiotherapy response was investigated. Two PI3K inhibitors were investigated and both BKM120 and GDC0980 effectively inhibited cellular and clonogenic growth in 6 HNSCC cells, both HPV-positive as well as HPV-negative. Despite targeted inhibition of the pathway and slight increase in DNA damage, PI3K inhibition did not show significant radiosensitization. Currently only one clinical trial is assessing the effectiveness of combining BKM120 with RT in HNSCC (NCT02113878) of which the results are eagerly awaited.

## Introduction

With almost 650,000 diagnoses and 330,000 deaths every year, head and neck squamous cell carcinoma (HNSCC) remains the sixth most prevalent cancer worldwide^1^. Tobacco and alcohol use are known risk factors for HNSCC as well as infection with human papillomavirus (HPV)^2,3^. Radiotherapy (RT) plays a prominent role in HNSCC treatment next to chemotherapy and surgery^4^. The 5-year survival proportion for locally advanced HNSCC is only 40–50% with locoregional recurrence remaining the predominant form of treatment failure, often linked to radioresistance of the tumour^5,6^. It is documented that activation of signal transduction pathways can lead to enhanced DNA repair in tumour cells resulting in a decreased efficiency of RT^7^.

One of the key pathways involved in resistance to therapies, including resistance to RT, is the phosphatidylinositol 3 kinase (PI3K)—protein kinase B (AKT)—mammalian target of rapamycin (mTOR) pathway^8^. This pathway is involved in three main mechanism of radioresistance namely tumour cell proliferation, hypoxia and intrinsic radioresistance^9^. Furthermore, it is known that this pathway has implications in classic tumorigenic processes such as apoptotic resistance, angiogenesis, invasion and metastases^10,11^. According to The Cancer Genome Atlas (TCGA), activating mutations in the *PIK3CA* gene are seen in 56% of HPV-positive and 34% of HPV- negative HNSCC^12,13^. Mutations in PTEN, AKT or the mTOR complex may lead to constitutive activation of the PI3K/AKT/mTOR pathway, resulting in cell growth and proliferation in the absence of nutrients^14,15^. Therefore, there is intense interest in molecular therapies targeting the PI3K/AKT/mTOR pathway^8^.

Multiple inhibitors of the PI3K/AKT/mTOR pathway have been developed, of which we selected two to study the efficacy in combination with RT in HNSCC cells^13,16^. Buparlisib, NVP-BKM120 (BKM120), is a novel, selective pan-class 1 PI3K inhibitor that works via reversible competition to ATP^17–19^. The anti-proliferative, pro-apoptotic and anti-angiogenic effects of BKM120 have been proven in a variety of tumour types, irrespective the *PIK3CA* status^20–22^. Currently six clinical trials are ongoing with BKM120 in HNSCC, of which only 1 involves combination with RT (NCT02113878). Next, the dual PI3K-mTOR inhibitor Apitolisib, GDC0980, is a small molecule inhibitor of class 1 PI3K and mTOR (mTORc1 and mTORc2) synthesized by substituting the indazole in GDC-0941 for a 2-aminopyrimidine^16,23^. Preclinical testing of GDC0980 showed attainable inhibition of the pathway, great potency against a wide variety of cell lines and inhibited tumour growth in vivo^24,25^.

In this study we investigated the response to a pan PI3K inhibitor, BKM120, and a dual PI3K/mTOR inhibitor, GDC0980, in three HPV-positive and three HPV-negative HNSCC cell lines alone and in combination with RT.

## Results

### The effect of PI3K inhibition alone or in combination with RT on cellular growth and survival

**Figure 1:**
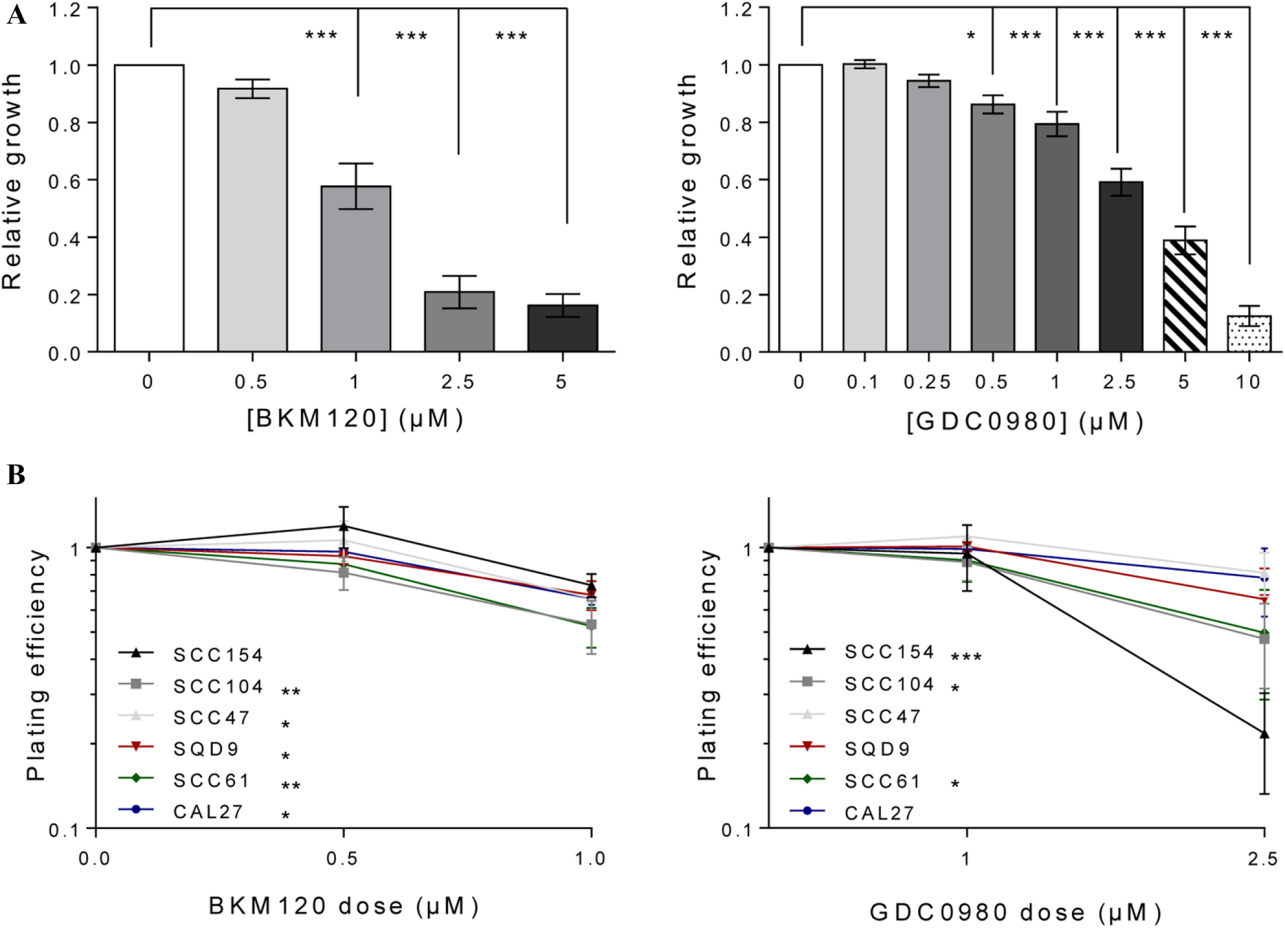
**(A)** Dose response curves of BKM120 (left) and GDC0980 (right). Cells were treated for 72 h with the indicated concentrations of BKM120 (left) and for 24 h with GDC0980 (right). Data is presented as the mean ± SEM of three HPV-positive cell lines (SCC154, SCC104 and SCC47) and three HPV-negative cell lines (SQD9, SCC61, CAL27) HNSCC cell lines of which 3 independent experiments were performed per cell line. One-way Anova analysis plus posthoc t-testing with Bonferroni correction showed the statistical significant differences. (**B)** The effect of PI3K inhibition monotherapy on plating efficiency of HNSCC cell lines. Colony assay of HNSCC cells treated with BKM120 for 72 h (left) or with GC0980 for 24 h (right). Colony formation was tested in three HPV-positive cell lines (SCC154, SCC104 and SCC47) and three HPV-negative cell lines (SQD9, SCC61, CAL27). Data are represented as the mean plating efficiency ± SEM for three independent performed experiments. One-way Anova analysis plus posthoc t-testing with Bonferroni correction showed the statistical significant differences.

The effect of PI3K inhibition on the growth and survival of HNSCC cells was investigated with the pan-PI3K inhibitor BKM120 and the dual PI3K and mTOR inhibitor GDC0980 by a short-term survival assay and colony assay (Fig. 1A,B). Both PI3K inhibitors BKM120 (0–5 µM) and GDC0980 (0–10 µM) effectively inhibited cellular growth dose-dependently in all HNSCC cells (Fig. 1A, Supplementary Fig. 1).

Based on the dose response curves, two concentrations for each of the inhibitors were selected for colony assay. BKM120 (0.5 and 1 µM incubated for 72 h) and GDC0980 (1 and 2.5 µM incubated for 24 h) monotherapy resulted in decreased colony formation at the highest treated concentrations. In addition, different cell lines showed differences in the sensitivity to the drug (Fig. 1B, Supplementary Fig. 2).

**Figure 2:**
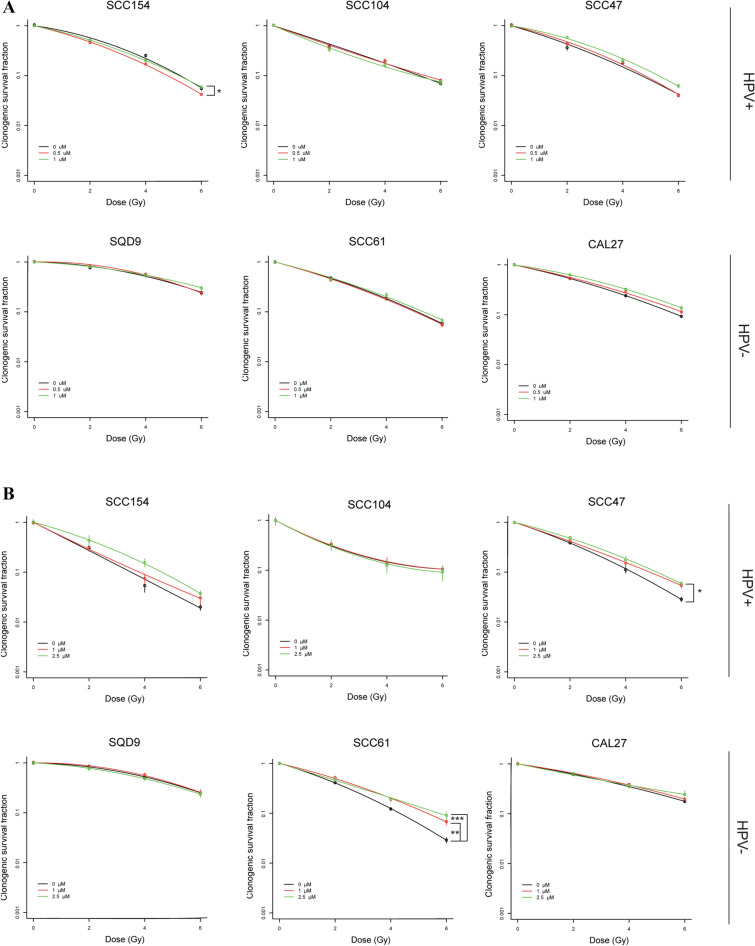
The effect of PI3K inhibition on the clonogenic survival of HNSCC cell lines. **(A)** Colony assay of HNSCC cells treated with BKM120. Cells were irradiated 2 h after drug exposure and the drug was removed 70 h after radiotherapy. **(B)** Colony assay of HNSCC cells treated with GDC0980. Cells were irradiated 2 h after drug exposure and the drug was removed 22 h after radiotherapy. Data are represented as the mean ± SEM for three independent performed experiments. The fitted curves were compared with Anova analysis to show the statistical significant differences.

BKM120 (0.5 and 1 µM) in combination with RT (given 2 h before RT for 72 h) did not enhance the RT response of the HPV-positive and HPV-negative HNSCC cells (Fig. 2A). Only in the HPV-positive cell line SCC154 a small radiosensitizing effect was seen at 0.5 µM (p = 0.02146). Also, GDC0980 (1 and 2.5 µM) in combination with RT (given 2 h before RT for 24 h) did not result in decreased survival of RT treated HNSCC cells (Fig. 2B). In some cell lines, SCC47 and SCC61, even an increase in survival was observed (p = 0.01989 for SCC47, p = 0.006706 and p = 3.692*10^–5^ for SCC61). To assess whether continuous PI3K inhibition is needed for radiosensitization, HNSCC cells were exposed to BKM120 or GDC0980 (0.1, 0.25 and 0.5 µM) in combination with RT (2 h before RT) for 2–3 weeks long. However, no radiosensitizing effect was observed (Supplementary Fig. 3). Here again, for some cell lines, there was a statistically significant increase in survival (p = 0.03756 and p = 0.04984 for CAL27, and p = 0.006371 for SCC61).

**Figure 3:**
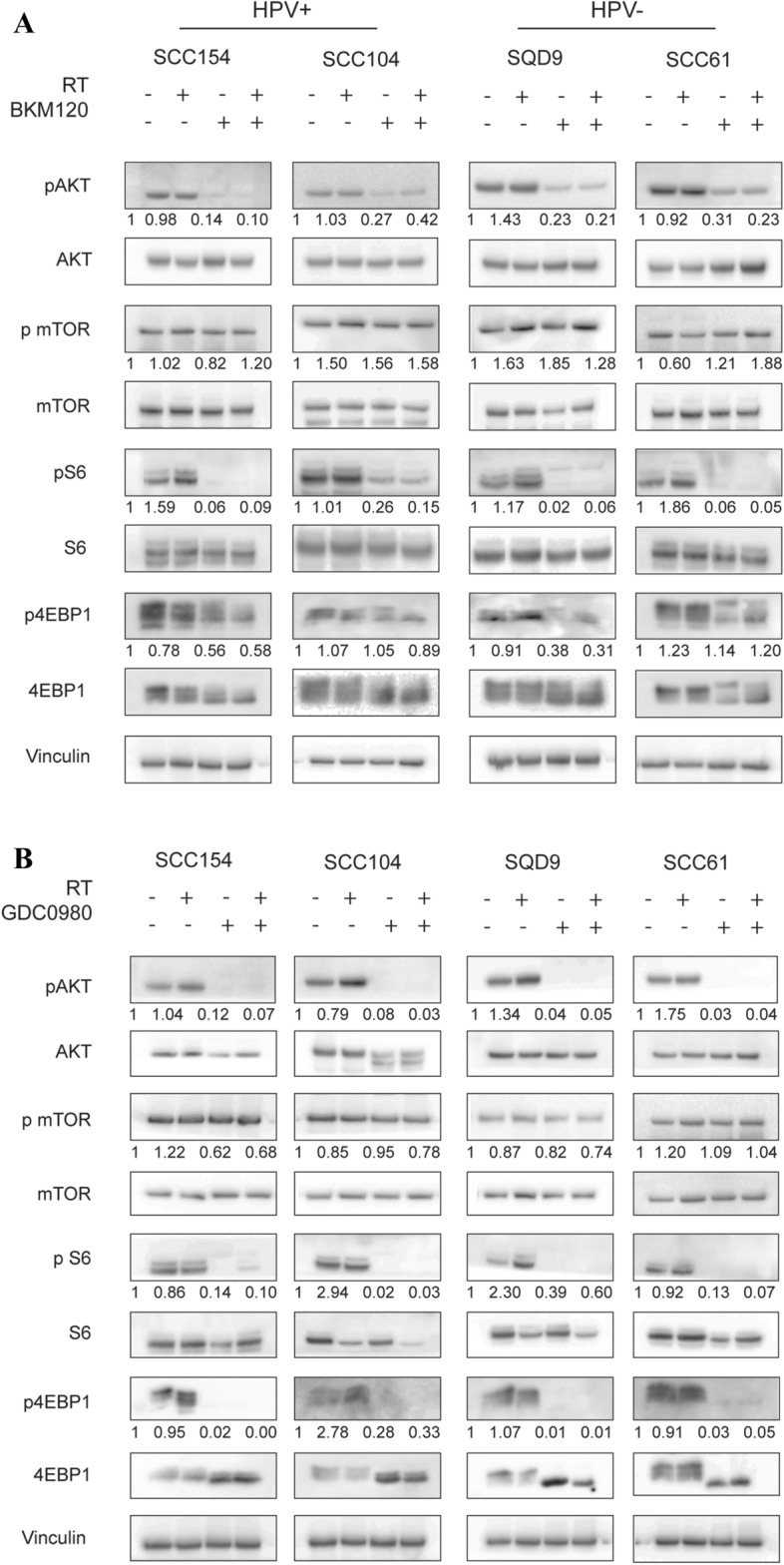
The effect of BKM120 and GDC0980 on the PI3K pathway. **(A)** Cells were treated with BKM120 (1 µM) 2 h before radiotherapy (6 Gy). Control condition cells are treated with equal concentrations of DMSO. After 24 h, total cell lysates were prepared to perform western blotting. **(B)** Cells were treated with GDC0980: 2.5 µM for HPV-positive cell lines (SCC154 and SCC104) and 1 µM for HPV-negative cells (SQD9 and SCC61). Control condition cells are treated with equal concentrations of DMSO. GDC0980 was administered 2 h before radiotherapy (6 Gy). After 24 h of drug treatment, total cell lysates were prepared to perform western blotting. Densitometry quantification was done and relative expression levels were corrected to the loading control and to the non-phosphorylated protein. Uncropped images are provided in the supplementary information.

### The effect of PI3K inhibition alone or in combination with RT on PI3K/AKT/mTOR pathway

The inhibitory effect of both inhibitors on the PI3K pathway was investigated by immunoblotting key proteins of the pathway in two HPV-positive (SCC154, SCC104) and two HPV-negative (SQD9, SCC61) HNSCC cell lines. Both BKM120 and GDC0980 treatment resulted in inhibition of the PI3K/AKT/mTOR pathway as can be seen by a reduction in phosphorylation of AKT, S6 and 4EBP1 compared to the untreated controls (Fig. 3). Inhibition of PI3K pathway by the PI3K inhibitors was seen in the monotherapy conditions as well as in the combination treatment condition (Fig. 3). A more effective inhibition of pAKT and p4EBP1 could be observed with the dual inhibitor.

### The effect of PI3K inhibition alone or in combination with RT on DNA damage response

**Figure 4:**
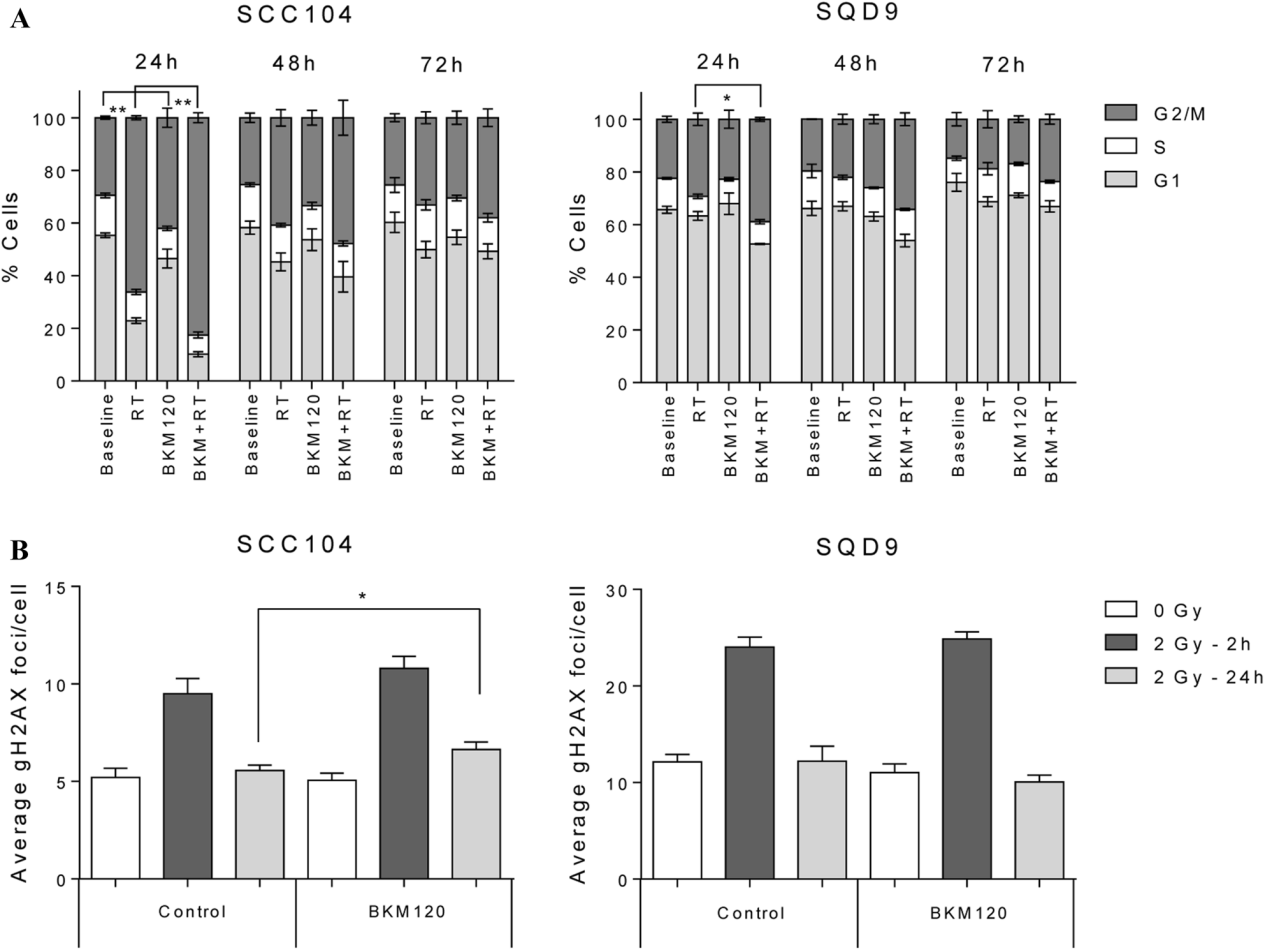
**(A)** The effect of BKM120 on the cell cycle. The percentage (%) of SCC104 HPV-positive (left) and SQD9 HPV-negative (right) HNSCC cells distributed in cell cycle phases after treatment for 24 h with 1 µM BKM120 and/or 6 Gy. Data are represented as the mean ± SEM for three independent performed experiments. One-way Anova plus posthoc t-testing with Bonferroni correction showed the statistical differences. **(B)** The effect of PI3K inhibition on the DNA damage response. **(A)** HPV-positive (SCC104) and HPV-negative (SQD9) cells were exposed to 1 µM of BKM120 and/or 6 Gy of radiotherapy to assess gH2AX foci. Fixation was done after 2 or 24 h to represent kinetics of DNA repair. Data are represented as the mean ± SEM for three independent performed experiments. T-testing showed the significant difference (p = 0.0454).

We assessed whether PI3K inhibition (1 µM BKM120) alone or in combination with RT (2 h after drug exposure) can induce alterations in cell cycle and gH2AX foci kinetics in HPV- positive SCC104 and HPV-negative SQD9 cells (Fig. 4A, B).

BKM120 monotherapy induced a significant G2/M arrest at 24 h compared to the baseline condition in the HPV-positive cells (p = 0.0058). When BKM120 was added to RT treatment, this resulted in a significant G2/M arrest after 24 h in both the HPV-positive (SCC104) and HPV-negative (SQD9) cells (p = 0.0021 and p = 0.0093 respectively) compared to the RT conditions. After 72 h the cells rendered back to the baseline condition (Fig. 4A).

As expected, a significant increase (twofold) in gH2AX foci was seen 2 h after RT alone in both cell lines (p < 0.0001) (Fig. 4B). The combination of BKM120 and RT did show residual gH2AX foci in the HPV-positive cell line at 24 h (p = 0.0454). Whereas in the HPV-negative cells no difference in gH2AX was detected between the RT treatment alone or in combination with BKM120 (Fig. 4B).

## Discussion

Since the relapse rate of locally advanced HNSCC remains very high despite progress made in surgical and radiotherapeutic techniques, molecular targeted drugs have become an appealing strategy to improve treatment response. Unfortunately very few targeted agents advanced to phase 3 clinical trials in combination with RT^26^. In this paper, we investigated the radiosensitizing potential of PI3K/AKT/mTOR pathway inhibitors in a panel of six HNSCC cell lines.

Although the PI3K pathway is the most frequently deregulated pathway in HNSCC, scientific literature is replete of contradictory data around the predictive value of *PIK3CA* status. Several studies showed that mutations in PIK3CA, which lead to PI3K/AKT activation, correlate with response to PI3K inhibition^11^. This in contrast to amplification of PI3K/AKT that has been associated with radioresistance^27^. Alpelisib, the first PI3K inhibitor approved by the FDA, is approved to treat breast cancer patients with PIK3CA-mutation^28^. The selectivity of Alpelisib for PIK3CA-mutated cells has also been seen in HNSCC cells by Keam et al*.*^29^. However, when we look at BKM120, the study of Kong et al*.* investigated the effect of BKM120 in *PIK3CA* hotspot mutated and wild type cell lines and it was concluded that BKM120 exhibit potent activity in both^30^. Since the value of *PIK3CA* status as predictive biomarker is still unknown according to Anizussaman et al*.* and we had limited availability of HNSCC cell lines with a *PIK3CA* mutation, we did not incorporate a *PIK3CA*-mutated cell line in our experimental set-up^15,31–35^.

Preclinical models showed that BKM120 has a strong anti-proliferative activity in more than 400 cancer cell lines^36^. This is in line with our study in which we see that both PI3K inhibitors showed significant inhibitory effects on proliferation and growth of all the tested cell lines, which were wildtype for PIK3CA. Although, it should be mentioned that the antiproliferative effects of the inhibitors were cell line dependent.

Next, we evaluated whether PI3K inhibition could increase the RT response in HNSCC cells. Although we could demonstrate effective inhibition of the PI3K/AKT/mTOR pathway, no radiosensitizing effect was observed despite testing different time schedules. This is in concordance with the study of Blas et al*.* in which different time schedules were tested and no significant difference in HNSCC was observed^37^. In addition, combining RT with BKM120 did not modify the radiation response and even reduced the effectiveness of BKM120 alone in inhibiting the PI3K/AKT/mTOR pathway^37^. In literature, other dual PI3K/mTOR inhibitors, namely PF-05212384, PF-04691502 and the β-sparing PI3K inhibitor GDC-0032, showed significant radiosensitizing effects both in vitro and in vivo in HNSCC^38–40^. Moreover, BKM120 demonstrated radiosensitizing effects in other cancer types^41–43^. However, the radiosensitizing effect of BKM120 in HNSCC has only been described mainly in vivo in two studies^44,45^. In these studies the tumour microenvironment seems to play an important role since the PI3K/AKT/mTOR pathway plays a role in O2-consumption thereby contributing to tumour hypoxia^46^. The importance of oxygenation has also been seen in the study of Burrows et al. where PI3K inhibition with GDC0941 in combination with RT reduced clonogenic survival only under anoxia conditions^47^. It has also been shown that BKM120 remodeled tumour vasculature and decreased hypoxia which could explain the radiosensitizing effects seen in vivo but not in vitro as in the study of Blas et al.^18,48,49^. Moreover, recent results published about the phase 1 clinical trial investigating the effect of BKM120 on palliative RT showed that BKM120 indeed reduced tumour hypoxia in NSCLC patients^50^. Studies using GDC0980 are limited to breast cancer and mesothelioma of which none combined GDC0980 with RT^51,52^.

The influence of the PI3K pathway on DSB repair and DNA damage response is documented in several studies and is one of the major factors for the hypothesis of combining PI3K inhibitors with RT^7,53–55^. In our study, the combination of BKM120 with RT resulted in a significant but limited presence of residual DNA damage in the HPV-positive cell line. The HPV-positive SCC104 cells treated with RT demonstrated less gH2AX foci than the HPV-negative HNSCC cells which can be ascribed to impairment in DSB repair in HPV-positive HNSCC cells due to decreased activity of both NHEJ and HRR and is in concordance with the knowledge that HPV-positive cells show increased sensitivity to RT^56–59^.

In line with our gH2AX data, limited but significant G2/M arrest was seen in the combination treatment arm for both HPV-positive and HPV-negative cells. After 72 h, the cells rendered back to baseline conditions in both the RT as well as the combination treatment condition due to further doubling of the cells and DNA repair. Comparing the combination treatment with RT alone, no residual additional differences were seen. G2/M arrest might result from persistent G2/M checkpoint activation caused by unrepaired double strand breaks and can have a protective function providing additional repair time for the cells before entering mitosis^56^. G2/M arrest after RT in HPV-positive cells is extensively documented and has been correlated with increased RT sensitivity of these cells^56,59,60^. PI3K inhibition-related G2/M arrest has been documented in glioblastoma and gastrointestinal carcinoma cells and can be explained by the fact that several important proteins in the regulation of the G2/M cell cycle checkpoint (e.g. Chk1, Wee1) have been identified as targets of PI3K^19,21,61–63^. Moreover, in PTEN-loss cells it was shown recently that AKT inhibition restored the relocalization of Chk1 in the nucleus, which rescued G2/M checkpoint activation so that DNA damage can be repaired^64^.

When we have a look at the results of clinical trials in HNSCC, the success of PI3K inhibitors is underwhelming with overall response rates of only 18%^18,65^. The low clinical durability and resistance to PI3K inhibitors have repeatedly been reported in literature^14^. The activation of rescue pathways can be a part of the answer to this^44,45,65,66^. In addition, the half maximum inhibitory concentration (IC50) of BKM120 to fully block all PI3K forms as monotherapy is high, leading to cellular toxicity via disrupting microtubule dynamics^11,21,67,68^. Moreover, toxicity to normal tissues has been observed with PI3K inhibitors due to broad-spectrum inhibition of multiple signalling pathways^7^. Finally, it is known that PI3K inhibition leads to cytostatic effects and are not cytotoxic which means that tumour regression is not achieved^7,32,65^. However, results of a randomised phase 2 study suggest that BKM120 combined with Paclitaxel could be an effective second-line treatment for patients with platinum-pretreated recurrent or metastatic HNSCC^69^. In addition, it was shown that patients with TP53 alterations, HPV-negative status, low mutational load, or high infiltration of TILs or CD8-positive cells derived survival benefit of the combination of BKM120 and Paclitaxel^70^. Recently, the BURAN phase 3 trial has been initiated to investigate this further. Of four clinical trials that are ongoing with BKM120 in HNSCC, only one (NCT02113878) is assessing the effectiveness in combination with RT of which the results are eagerly awaited.

In conclusion, we showed that the both PI3K inhibitors, BKM120 and GDC0980, induced antiproliferative effects and effectively inhibited the pathway though they did not enhance the RT response in HNSCC cells.

## Materials and methods

### Cell culture

The SCC154 cell line was purchased from the German collection of micro-organisms and cell cultures (DSMZ, Germany). Cell lines SCC104 and SCC47 were granted from Dr. Carey Thomas (Michigan University). All three HPV-positive cells lines were cultured and maintained in minimum essential media (MEM, Thermofisher scientific) supplemented with 10% fetal bovine serum (FBS), 1% nonessential amino acids and 1% L-glutamine. The SQD9, SCC61 and CAL27 cell lines were a generous gift of Dr. A. Begg, the Netherlands Cancer Institute (Amsterdam, the Netherlands). These four HPV-negative cell lines were cultured and maintained in Dulbecco’s Modified Eagle’s Medium (DMEM, Thermofisher scientific) containing 10% FBS and 1% sodium pyruvate (Life technologies). Cells were incubated at 37 °C and passaged via trypsinization. All cell lines were authenticated by ATCC via short-tandem repeat profiling.

All cell lines were wild-type for exon 5, exon 6, exon 10 and exon 21 of the *PIK3CA* gene, determined by sanger-sequencing. These regions incorporate the hot spot mutations in both the helical domain (E545K) and the kinase domain (H1047R)^71,72^. Three polymorphisms were detected in exon 10: rs 121913273 only in SCC61, and both rs 587777795 and rs 121913274 in cell lines SCC154, SQD9, SCC61 and CAL27.

### Chemicals

Buparlisib (NVP-BKM120) and Apitolisib (GDC0980) were bought from Selleck Chemicals (Houston, TX, USA) and a stock solution of 10 mM was made in dimethylsulfoxide (DMSO).

### Sulforhodamine B assay

To evaluate the drug’s effects on cell survival and proliferation, cells were seeded in 96-well plates. After attachment overnight on 37 °C, cells were treated with drug concentrations of 0; 0.5; 1; 2.5; 5 µM of BKM120 and 0; 0.1; 0.25; 0.5; 1; 2.5; 5; 10 µM of GDC0980. Two hours after drug administration plates were irradiated with doses of 0, 2, 4 or 6 Gy using a Baltograph (199 kV, 15 mA, Balteau NDT, Belgium). The drug was replaced by fresh full medium after 72 h (h) for BKM120 and 24 h for GDC0980. The time of exposure has been based on pilot studies^32,68,73,74^. One week after seeding, cells were fixed and stained with sulforhodamine B (SRB, Sigma-Aldrich). The incorporated SRB was liberated from cells in a tris-base solution, and optical densities were determined at 570 nm. Cell survival was determined as the relative absorption of SRB in treated wells in comparison to controls.

### Colony assay

Cells were seeded in six-well plates and after attachment overnight, cells were treated with 0.5 and 1 µM of BKM120 and 1 or 2.5 µM of GDC0980. Two hours after drug exposure cells were irradiated with doses of 0, 2, 4 or 6 Gy. After a 14–21 day incubation period, colonies were stained with 0.4% crystal violet and survival fractions were determined by counting 50 cells or more with a ColCount (Oxford Optronix, Oxford, United Kingdom) as described^75^. Data was normalized to the plating efficiency of unirradiated, non-drug treated controls and curves were fitted using the LQ model as described^37^. Statistical differences in LQ curves were tested with package CFassay in R studio (RStudio: Integrated Development for R. RStudio, Inc., Boston, MA, USA).

### Western blotting

For immunoblotting cells were treated with 1 µM of BKM120 and for GDC0980, HPV-positive cells were exposed to 2.5 µM and HPV-negative cells to 1 µM. Drug concentrations for western blotting were chosen according the sensitivity of the cell lines to the inhibitors. Control conditions were treated with equal concentrations of DMSO. Two hours after drug exposure cells were irradiated with 6 Gy. Twenty-four hours after drug administration, cells were lysed in 1× RIPA buffer containing protease and phosphatase inhibitors (Roche). Protein concentrations were determined using Bradford method with Albumin Bovine Serum (BSA, 1 µg/µl, Sigma-Aldrich) as described^76^. Equal amounts of protein (15 µg) were separated on NUPAGE gels (Thermofisher scientific). After electrophoresis, proteins were transferred onto a polyvinylidene fluoride membrane (Biorad) and the membranes were blocked with 5% non-fatty dry milk. The blots were incubated overnight on 4 °C with primary antibodies for AKT (#9272), phospho-AKT Ser 473 (#9271), mTOR (#2972), phospho-mTOR ser2448 (#2971), p70S6 (#9202), phospho-p70S6 (#9205), 4EBP1 (#9452) and phospho-4EBP1 (#9459) from Cell signalling technology, Vinculin (Sigma-Aldrich) was used as loading control. After incubation with a secondary antibody, visualization was performed with enhanced chemiluminescence reagent (ECL Ultra, Perkin Elmer) using a chemiluminescence imager (c600 Azure Biosystems, CA, USA). Densitometry analysis was performed using ImageJ2 software. To avoid false results, all densitometry values were normalized to the loading control and according total protein. Thereafter, relative values were calculated by dividing each condition by the control condition, meaning no RT and no inhibitor treatment.

### Cell cycle analysis

Cells were seeded and after adherence, cells were treated with 1 µM of BKM120 and irradiated with 6 Gy 2 h after drug exposure. At the indicated timepoints cells were fixed with 70% ethanol and stained with 10 µg/ml propidium iodide containing 100 µg/ml RNAse A as described^77^. Cells cycle distribution was assessed by FACSverse (BD Biosciences, CA, USA).

### Immunofluorescence

As described^77^, cells were plated on µClear 96-well plates (Greiner Bio-one) and treated with 1 µM of BKM120 and irradiated with 2 Gy 2 h after drug administration. Samples were fixed 4 h and 24 h after drug exposure with 4% paraformaldehyde. After permeabilization with methanol, the cells were stained with primary antibody against gH2AX (ser 139) (clone JBW301 Millipore). This was followed by a staining with secondary antibody conjugated to FITC (Life Technologies). The nuclei were counterstained with DAPI (Sigma Aldrich). Immunofluorescence images were acquired with In Cell Analyzer 2000 (GE Healthcare, IL, USA).

### Statistical analysis

All experiments were conducted in triplicate at different time points. Statistical analysis was performed two-sided using GraphPad Prism version 5.00 for Windows (GraphPad Software, La Jolla California, SD, USA). All tests were considered as statistically significant for p < 0.05 (*), p < 0.01 (**) and p < 0.001 (***). In the figures, each value is represented as the mean ± SEM.

## Supplementary information

Supplementary Information.
