# Supplementary material for: The influence of PI3K inhibition on the radiotherapy response of head and neck cancer cells
Source: Sci Rep. 2020 Oct 1;10:16208. doi: 10.1038/s41598-020-73249-z (PMC7529775; doi:10.1038/s41598-020-73249-z)
Supplement: Supplementary file 1 — Supplementary Information. [file 41598_2020_73249_MOESM1_ESM.pdf]

Supplementary Information

The influence of PI3K inhibition on the radiotherapy response of head and neck cancer cells

Mary Glorieux [1], Rüveyda Dok [1], Sandra Nuyts [1,2,*]

[1] Laboratory of Experimental Radiotherapy, Department of Oncology, KU Leuven, University of Leuven, 3000 Leuven, Belgium

[2] Department of Radiation Oncology, Leuven Cancer Institute, UZ Leuven, 3000 Leuven, Belgium

Corresponding author:
Sandra Nuyts
UH Leuven
3000 Leuven
Belgium
Tel: +3216347600
Fax: +3216347623
E-mail: sandra.nuyts@uzleuven.be

Supplementary Figure 1

A

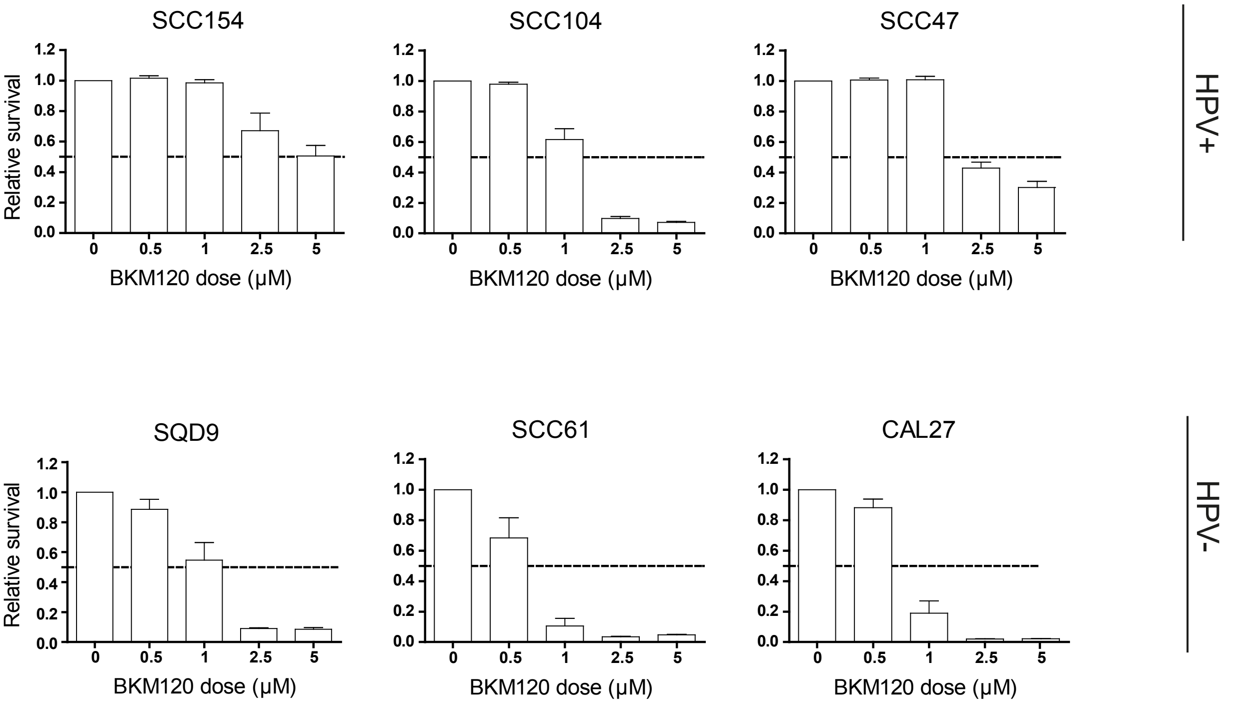

B

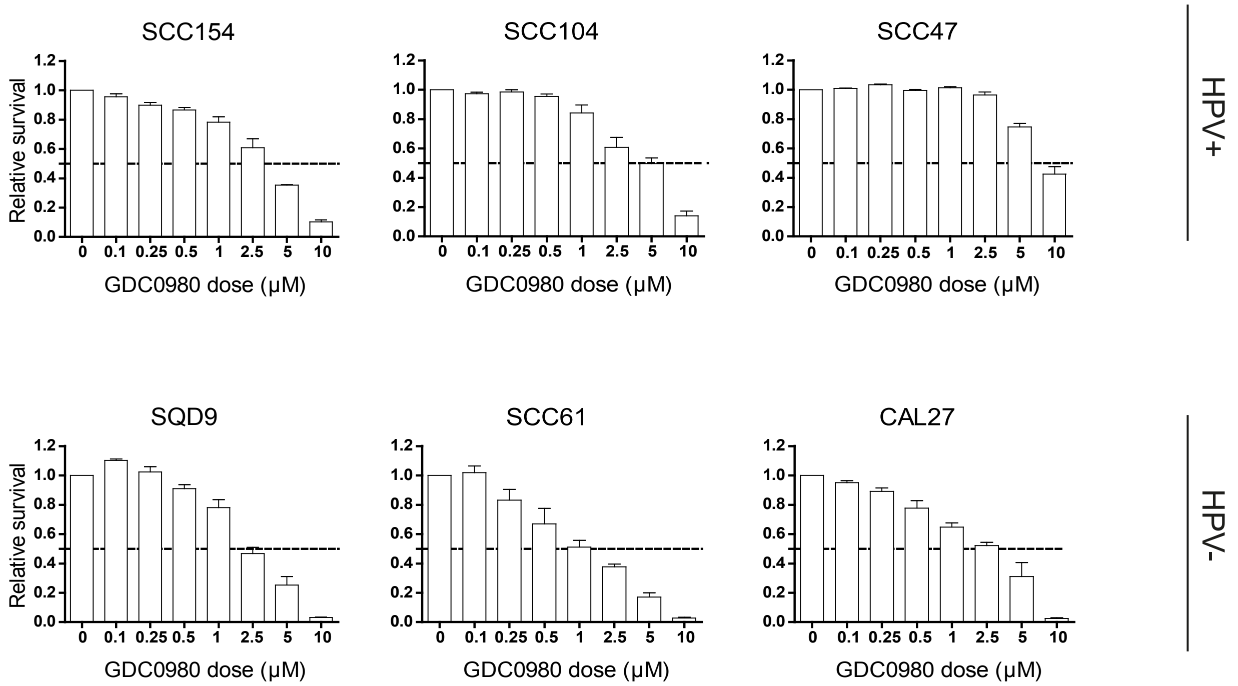

Supplementary Figure 2

A

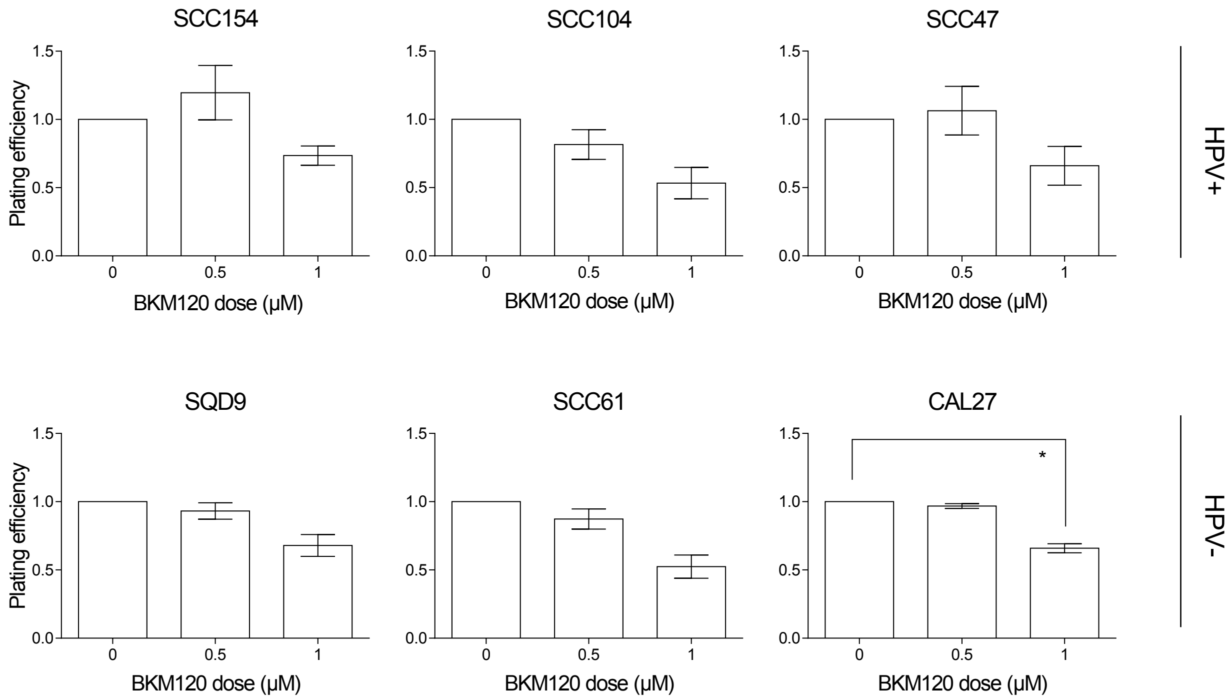

B

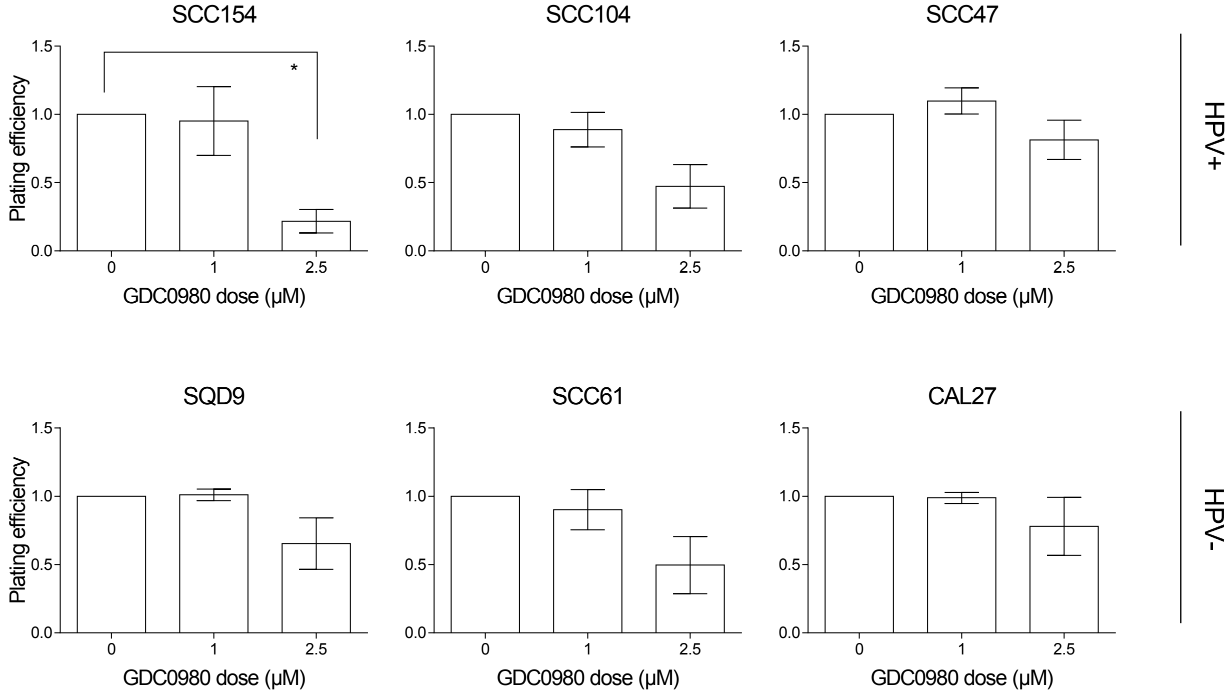

Supplementary Figure 3

A

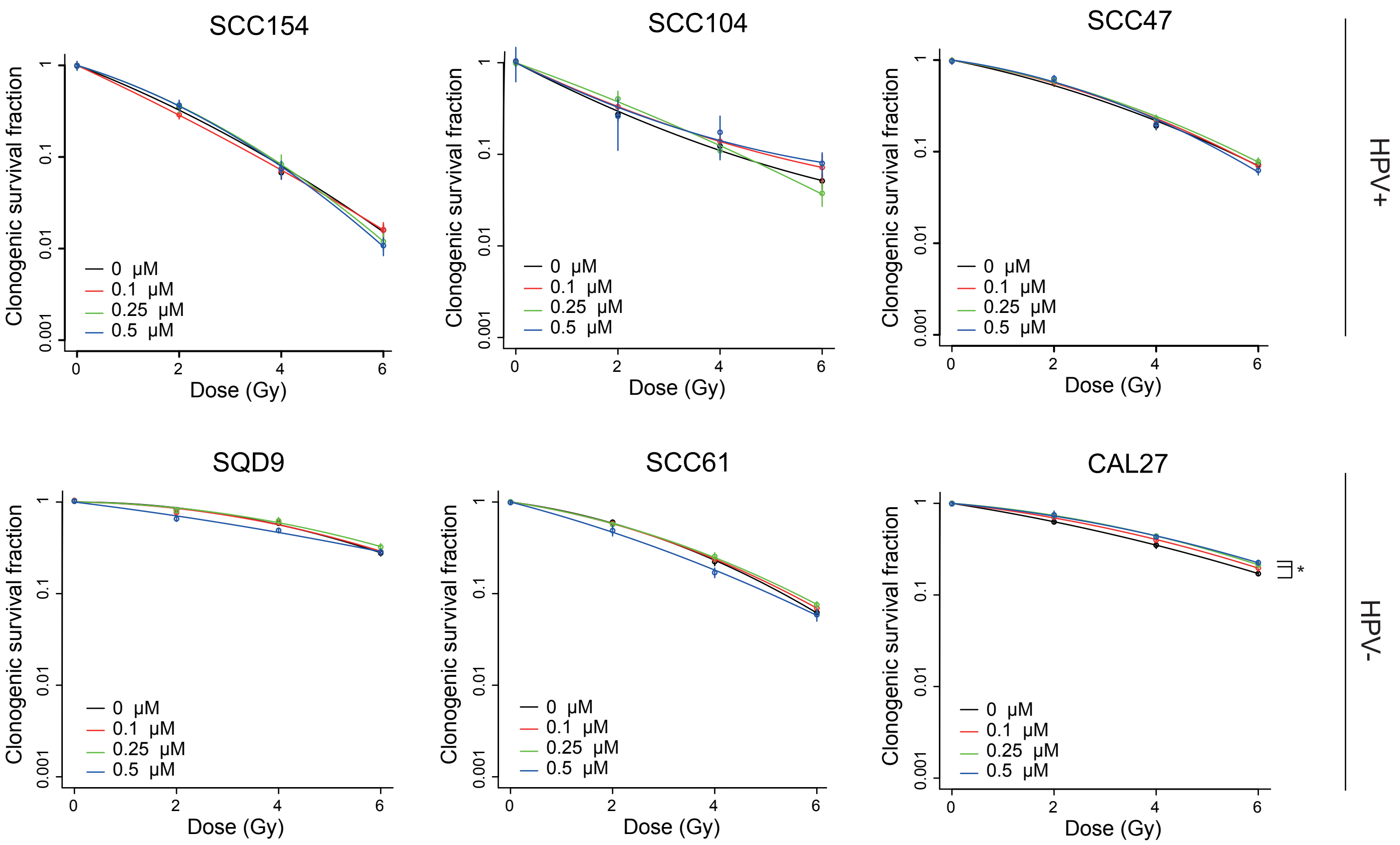

B

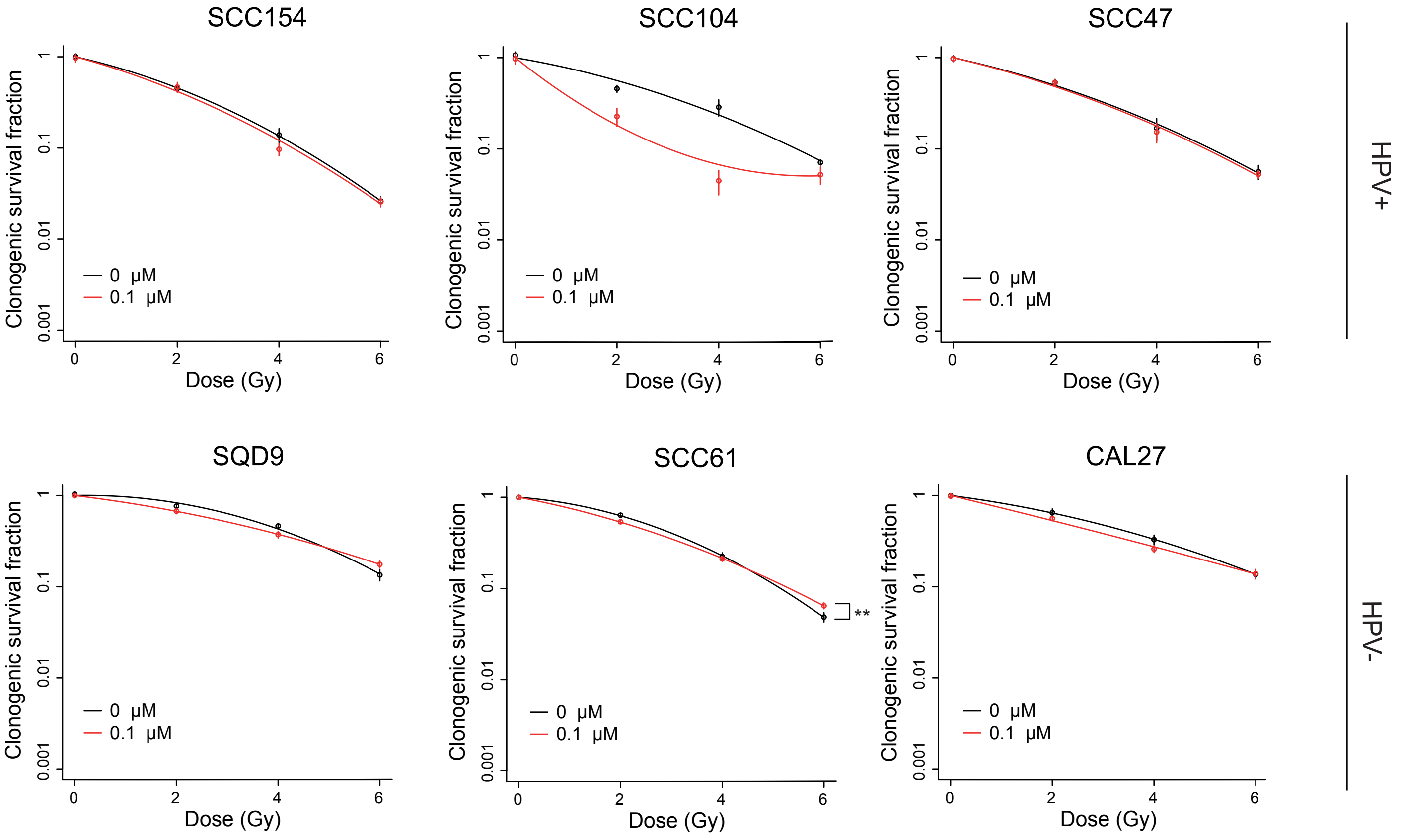

Supplementary Material 1

A

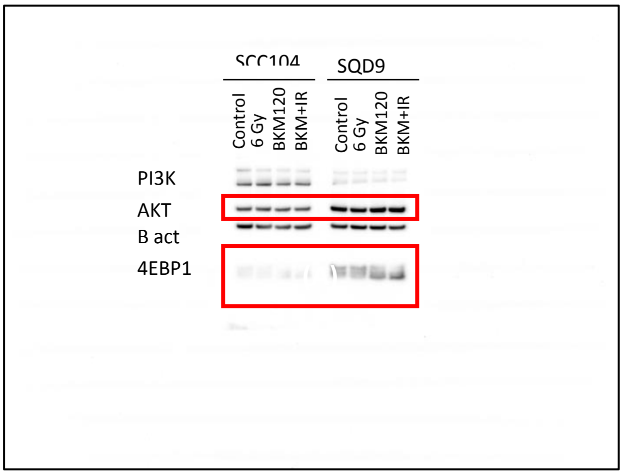

B

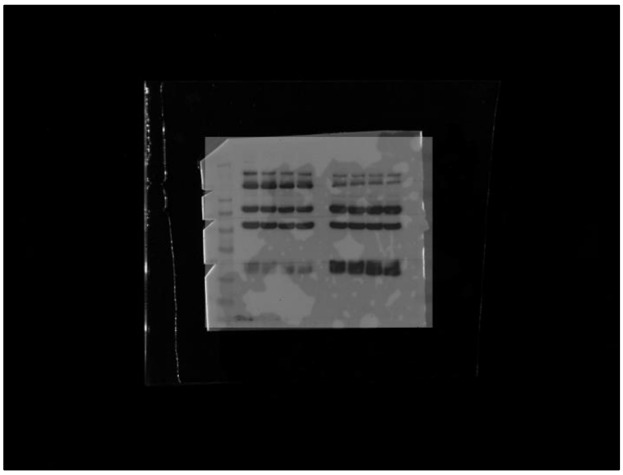

C

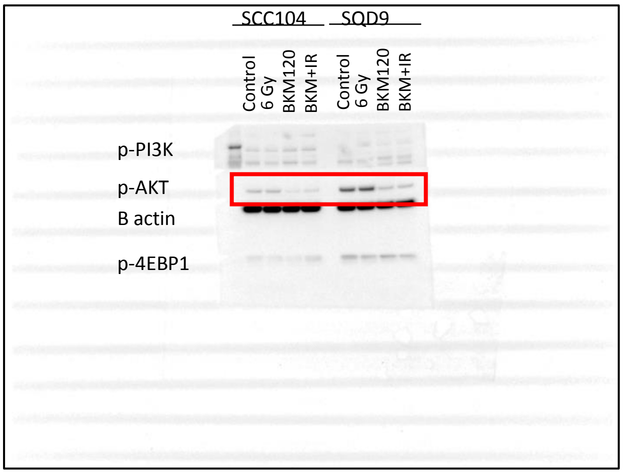

D

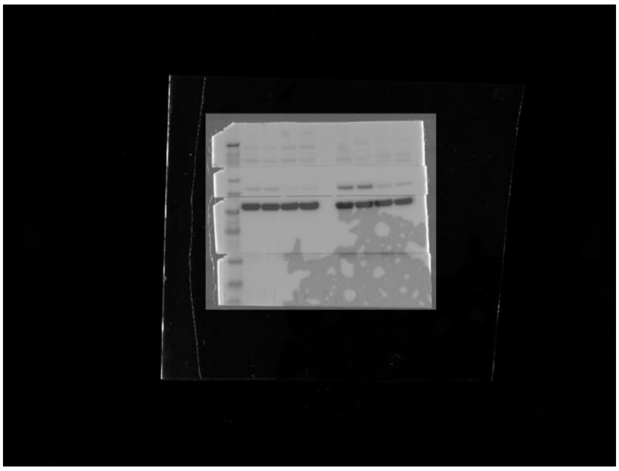

E

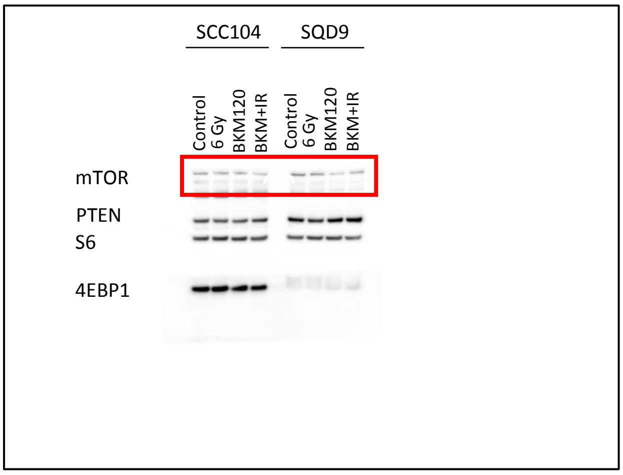

F

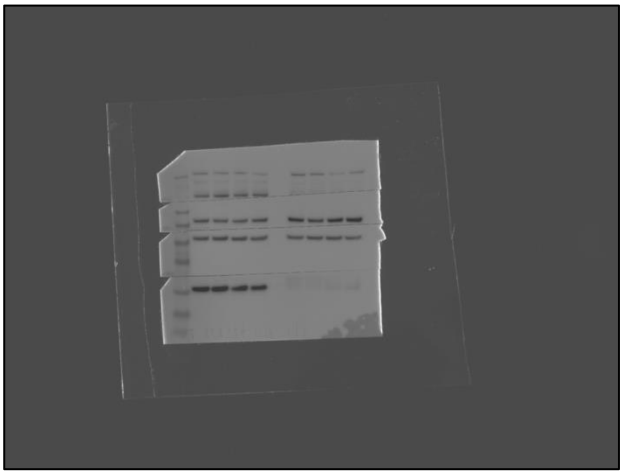

G

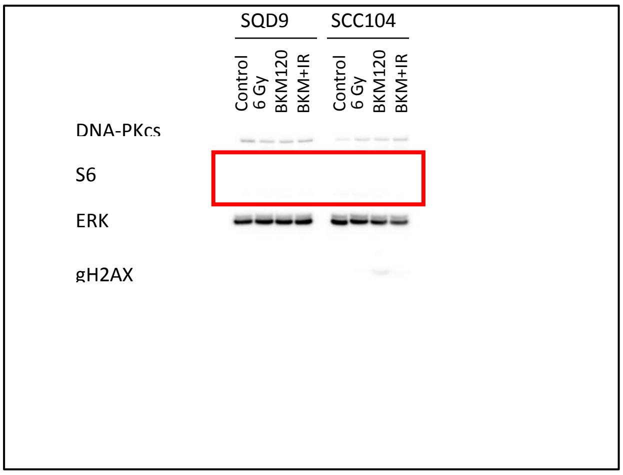
SQD9
SCC104
Control
6 Gy
BKM120
BKM+IR
Control
6 Gy
BKM120
BKM+IR
DNA-PKcs
S6
ERK
gH2AX

H

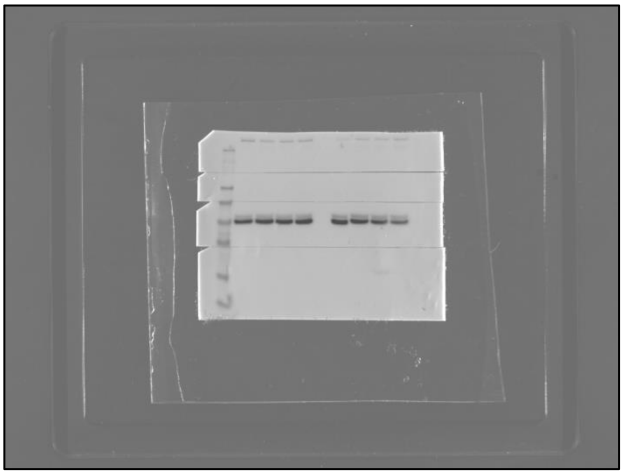

I

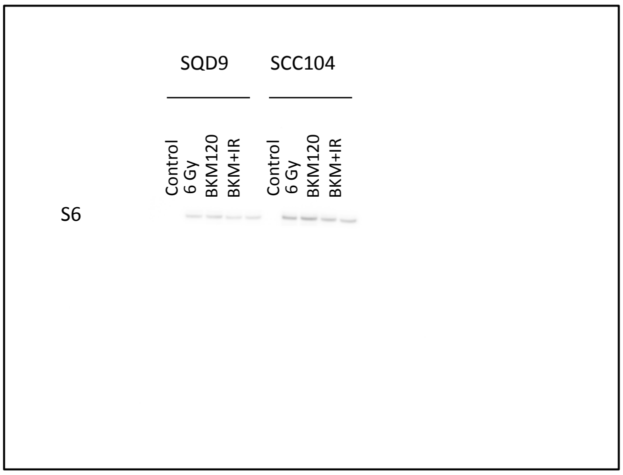
SQD9
SCC104
Control
6 Gy
BKM120
BKM+IR
Control
6 Gy
BKM120
BKM+IR
S6

J

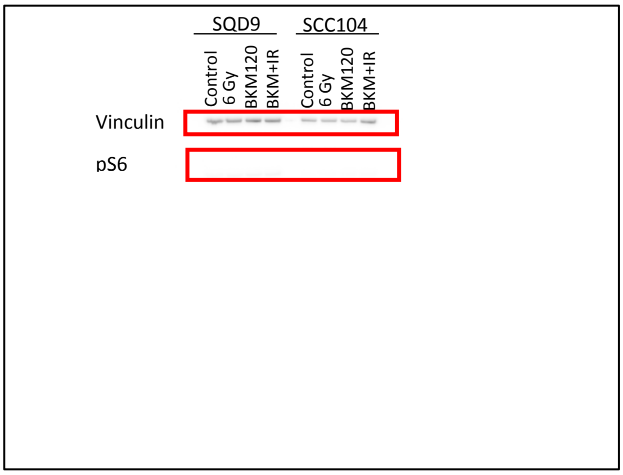
SQD9
SCC104
Control
6 Gy
BKM120
BKM+IR
Control
6 Gy
BKM120
BKM+IR
Vinculin
pS6

K

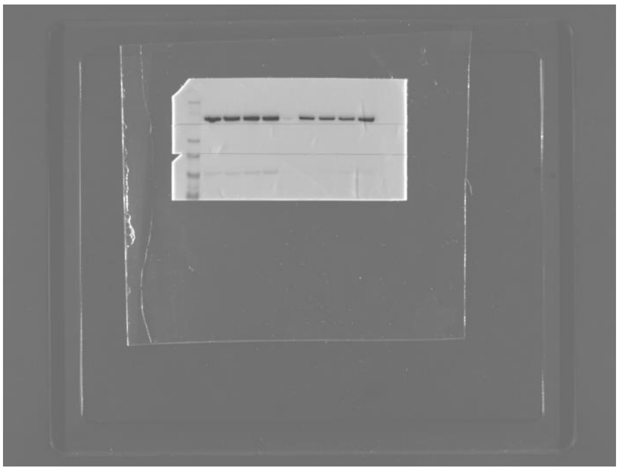

L

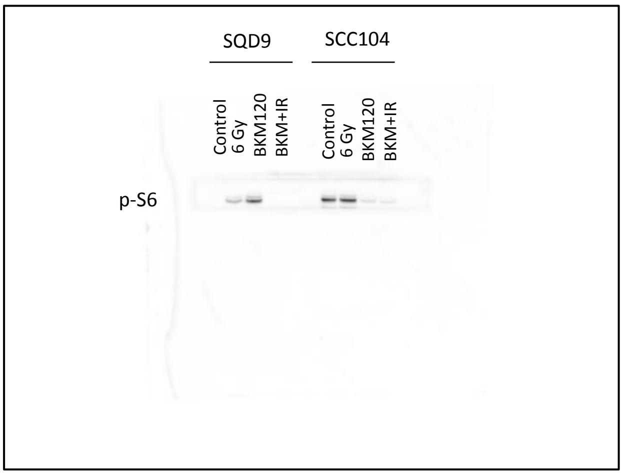
SQD9
SCC104
Control
6 Gy
BKM120
BKM+IR
Control
6 Gy
BKM120
BKM+IR
p-S6

M

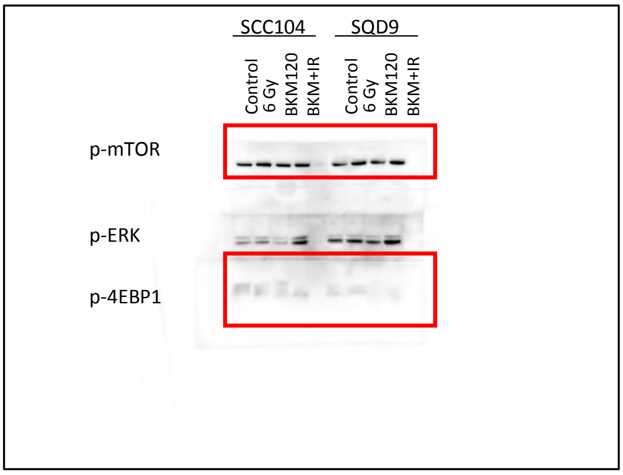

N

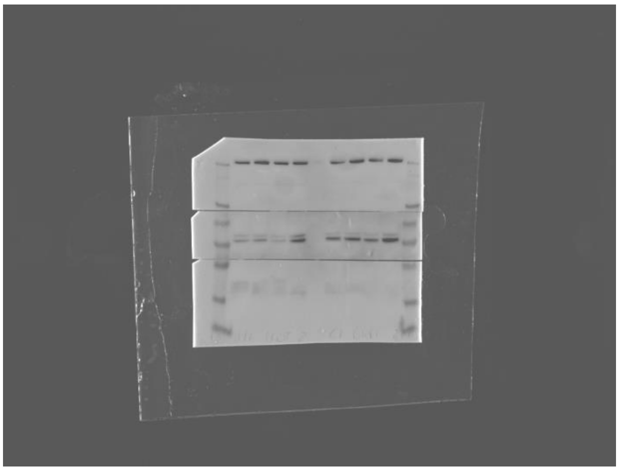

Supplementary Material 1. Uncropped western blot images of the head and neck cancer cell lines SCC104 and SQD9 used in Figure 3A. (A) Uncropped western blot of the cell lines SCC104 (lane 2-5) and SQD9 (lane 7-10) treated with vehicle, radiotherapy (6 Gy), BKM120 (1 µM) or the combination of radiotherapy and BKM120 (6 Gy and 1µM) respectively (left to right). From top to bottom, the expression of PI3K, AKT, b actin and 4EBP1 are visible in this blot, but only AKT and 4EBP1 are used in Figure 3A (red box). **(B)** Overlay image with protein ladder of the uncropped western blot of Supplementary Figure 2A (SeeBlue^{TM}, Thermofisher). **(C)** Uncropped western blot of the cell lines SCC104 (lane 2-5) and SQD9 (lane 7-10) treated with vehicle, radiotherapy (6 Gy), BKM120 (1 µM) or the combination of radiotherapy and BKM120 (6 Gy and 1µM) respectively. The expression of phospho-PI3K, phospho-AKT, b actin and phospho-4EBP1 are visible in this blot, but only phospho-AKT is used in Figure 3A. **(D)** Overlay image with protein ladder of the uncropped western blot of Supplementary Figure 2C (SeeBlue^{TM}, Thermofisher). **(E)** Uncropped western blot of the cell lines SCC104 (lane 2-5) and SQD9 (lane 7-10) treated with vehicle, radiotherapy (6 Gy), BKM120 (1 µM) or the combination of radiotherapy and BKM120 (6 Gy and 1µM) respectively. The expression of mTOR, PTEN, S6 and 4EBP1 are visible in this blot, but only mTOR is used in Figure 3A. **(F)** Overlay image with protein ladder of the uncropped western blot of Supplementary Figure 2E (SeeBlue^{TM}, Thermofisher). **(G)** Uncropped western blot of head and neck cancer cell lines SQD9 (lane 2-5) and SCC104 (lane 7-10) treated with vehicle, radiotherapy (6 Gy), BKM120 (1 µM) or the combination of radiotherapy and BKM120 (6 Gy and 1µM) respectively. The expression of DNA-PKcs, S6, ERK and gH2AX are visible in this blot, but only S6 is used in Figure 3A. **(H)** Overlay image with protein ladder of the uncropped western blot of Supplementary Figure 2G (SeeBlue^{TM}, Thermofisher). **(I)** Same blot as panel G and H that had been developed separately to get a good visualization of the protein S6. **(J)** Uncropped western blot of head and neck cancer cell lines SQD9 (lane 2-5) and SCC104 (lane 7-10) treated with vehicle, radiotherapy (6 Gy), BKM120 (1 µM) or the combination of radiotherapy and BKM120 (6 Gy and 1µM) respectively. The expression of vinculin and phospho-S6 are visible in this blot are used in Figure 3A. **(K)** Overlay image with protein ladder of the uncropped western blot of Supplementary Figure 2 panel J (SeeBlue^{TM}, Thermofisher). **(L)** Same blot as panel J had been developed separately to get a good visualization of the protein phospho-S6. **(M)** Uncropped western blot of head and neck cancer cell lines SCC104 (lane 2-5) and SQD9 (lane 7-10) treated with vehicle, radiotherapy (6 Gy), BKM120 (1 µM) or the combination of radiotherapy and BKM120 (6 Gy and 1µM) respectively. The expression of phospho-mTOR, phospho-ERK and phospho-4EBP1 are visible in this blot, but only phospho-mTOR and phospho-4EBP1 are used in Figure 3A. **(N)** Overlay image with protein ladder of the uncropped western blot of Supplementary Figure 2 panel M (SeeBlue^{TM}, Thermofisher).

Supplementary Material 2

A

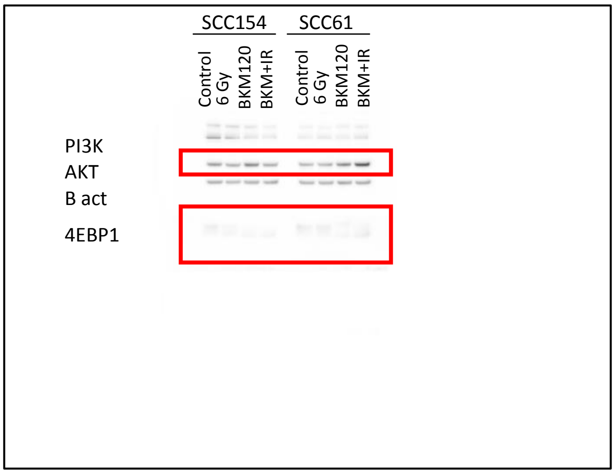

B

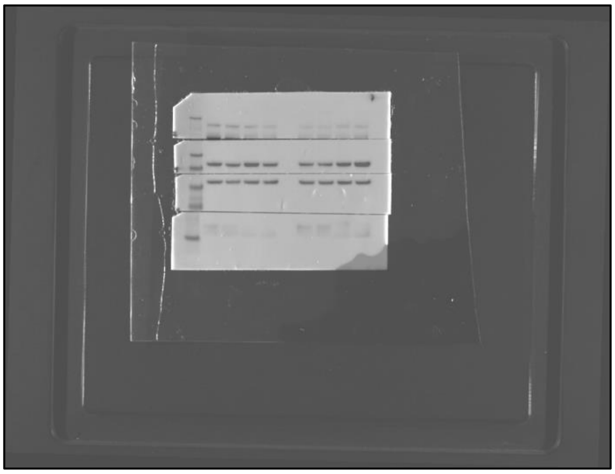

C

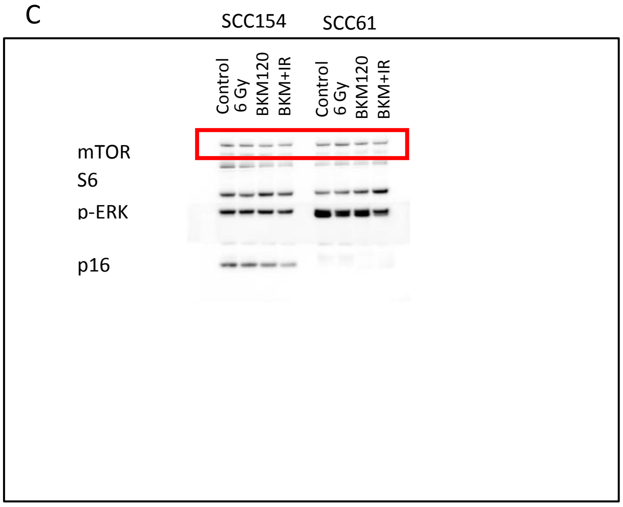

D

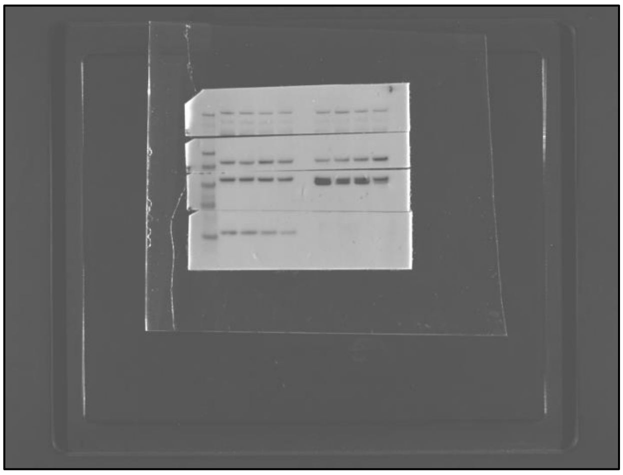

E

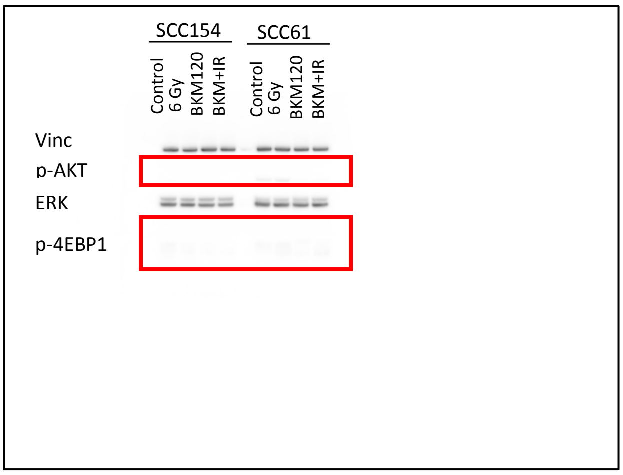

F

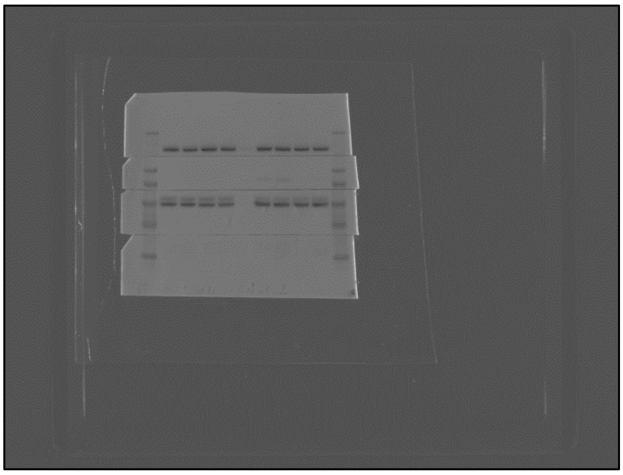

G

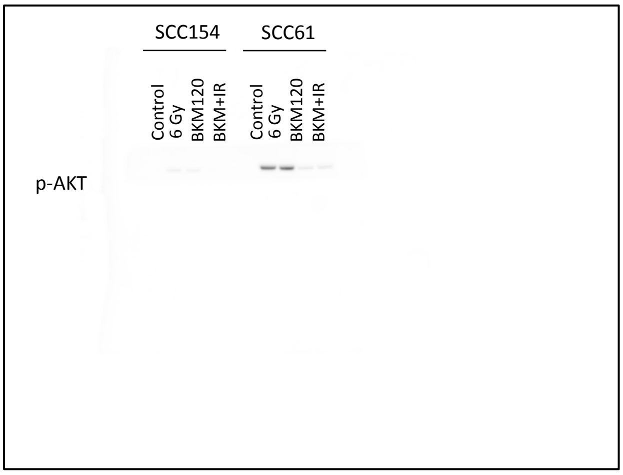
SCC154
SCC61
Control
6 Gy
BKM120
BKM+IR
Control
6 Gy
BKM120
BKM+IR
p-AKT

H

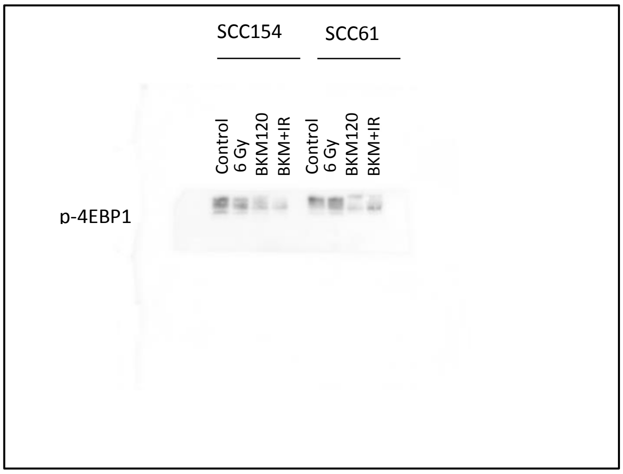
SCC154
SCC61
Control
6 Gy
BKM120
BKM+IR
Control
6 Gy
BKM120
BKM+IR
p-4EBP1

I

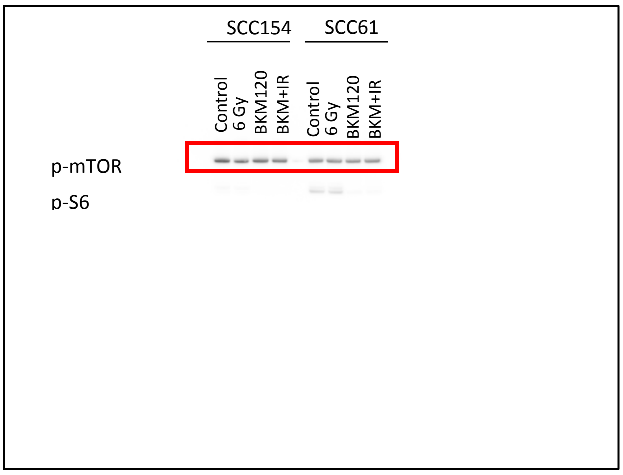
SCC154
SCC61
Control
6 Gy
BKM120
BKM+IR
Control
6 Gy
BKM120
BKM+IR
p-mTOR
p-S6

J

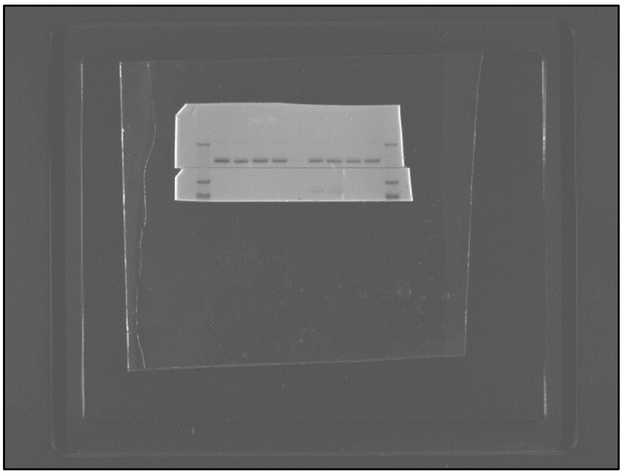

K

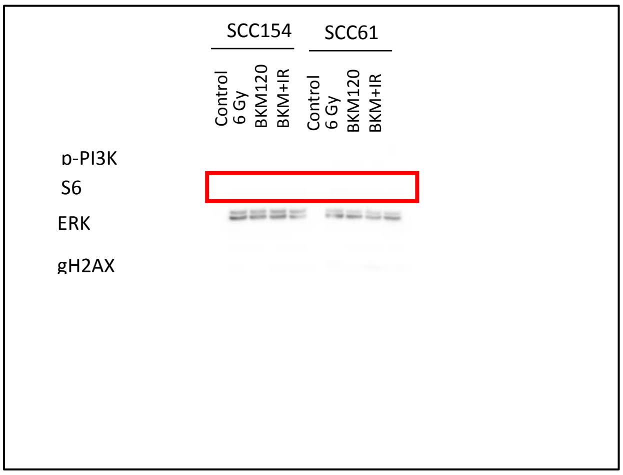
SCC154
SCC61
Control
6 Gy
BKM120
BKM+IR
Control
6 Gy
BKM120
BKM+IR
p-PI3K
S6
ERK
gH2AX

L

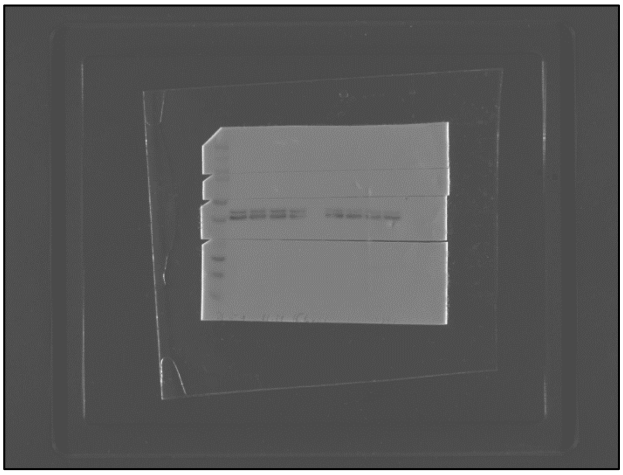

M

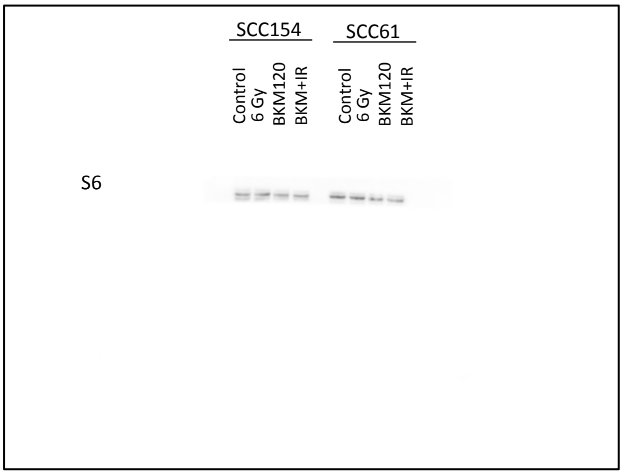

N

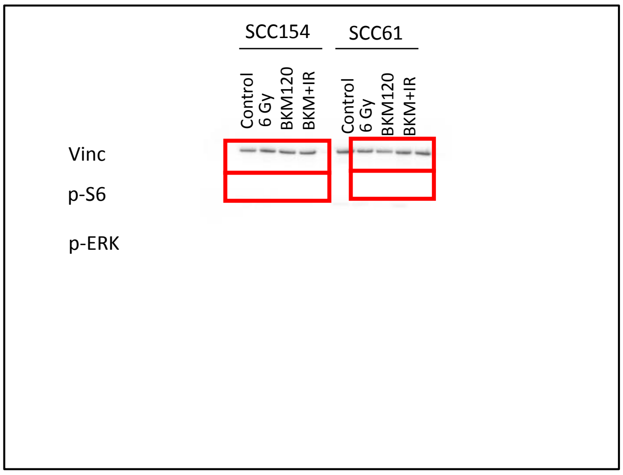

O

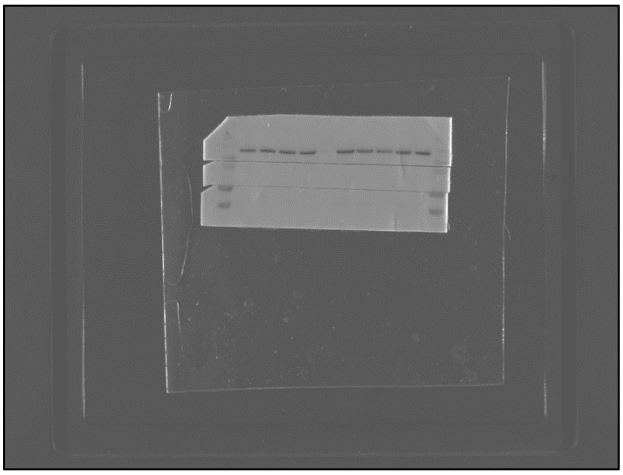

P

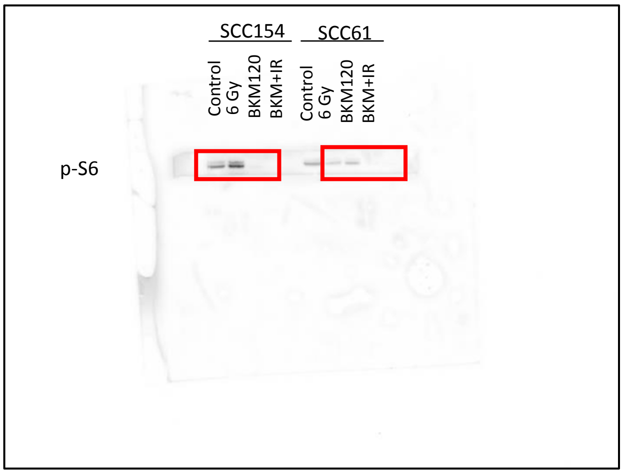

Supplementary Material 2. Uncropped western blot images of the head and neck cancer cell lines SCC154 and SCC61 used in Figure 3A. (A) Uncropped western blot of head and neck cancer cell lines SCC154 (lane 2-5) and SCC61 (lane 7-10) treated with vehicle, radiotherapy (6 Gy), BKM120 (1 µM) or the combination of radiotherapy and BKM120 (6 Gy and 1µM) respectively (left to right). From top to bottom, the expression of PI3K, AKT, b actin and 4EBP1 are visible in this blot, but only AKT and 4EBP1 are used in Figure 3A (indicated with the red box). **(B)** Overlay image with protein ladder of the uncropped western blot of Supplementary Figure 3A. **(C)** Uncropped western blot of head and neck cancer cell lines SCC154 (lane 2-5) and SCC61 (lane 7-10) treated with vehicle, radiotherapy (6 Gy), BKM120 (1 µM) or the combination of radiotherapy and BKM120 (6 Gy and 1µM) respectively. The same blot was used as in panel A. The expression of mTOR, S6, phospho-ERK and p16 are visible here, but only mTOR is used in Figure 3A. **(D)** Overlay image with protein ladder of the uncropped western blot of Supplementary Figure 3C. **(E)** Uncropped western blot of head and neck cancer cell lines SCC154 (lane 2-5) and SCC61 (lane 7-10) treated with vehicle, radiotherapy (6 Gy), BKM120 (1 µM) or the combination of radiotherapy and BKM120 (6 Gy and 1µM) respectively. The expression of vinculin, phospho-AKT, ERK and phospho-4EBP1 are visible here, but only phospho-AKT and phospho-4EBP1 are used in Figure 3A. **(F)** Overlay image with protein ladder of the uncropped western blot of Supplementary Figure 3E. **(G)** Second part of the blot in panel E and F had been developed separately to get a good visualization of the protein phospho-AKT. **(H)** Last part of the blot shown in panel E and F had been developed separately to get a good visualization of the protein phospho-4EBP1. **(I)** Uncropped

western blot of head and neck cancer cell lines SCC154 (lane 2-5) and SCC61 (lane 7-10) treated with vehicle, radiotherapy (6 Gy), BKM120 (1 µM) or the combination of radiotherapy and BKM120 (6 Gy and 1µM) respectively. The same blot was used as in panel E. The expression of phospho-mTOR and phospho-S6 are visible here, but only phospho-mTOR is used in Figure 3A. **(J)** Overlay image with protein ladder of the uncropped western blot of Supplementary Figure 3 panel I. **(K)** Uncropped western blot of head and neck cancer cell lines SCC154 (lane 2-5) and SCC61 (lane 7-10) treated with vehicle, radiotherapy (6 Gy), BKM120 (1 µM) or the combination of radiotherapy and BKM120 (6 Gy and 1µM) respectively. The expression of phospho-PI3K, S6, ERK and gH2AX are visible here, but only S6 is used in Figure 3A. **(L)** Overlay image with protein ladder of the uncropped western blot of Supplementary Figure 3 panel K. **(M)** Same blot as panel K and L that had been developed separately to get a good visualization of the protein S6. **(N)** Uncropped western blot of head and neck cancer cell lines SCC154 (lane 2-5) and SCC61 (lane 8-11) treated with vehicle, radiotherapy (6 Gy), BKM120 (1 µM) or the combination of radiotherapy and BKM120 (6 Gy and 1µM) respectively. The expression of vinculin, phospho-S6 and phospho-ERK are visible here, but only vinculin and phospho-S6 are used in Figure 3A. **(O)** Overlay image with protein ladder of the uncropped western blot of Supplementary Figure 3 panel N. **(P)** The bottom part of the blot shown in panel N and O had been developed separately to get a good visualization of the protein phospho-S6. For the ladder (SeeBlue^{TM}, Thermofisher) was used for all western blots.

Supplementary Material 3

A

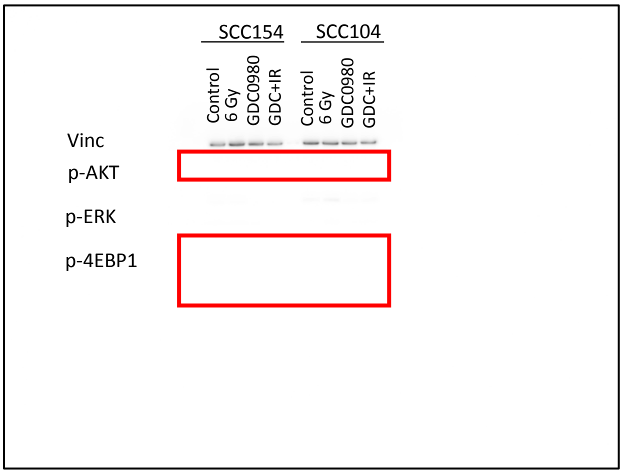

B

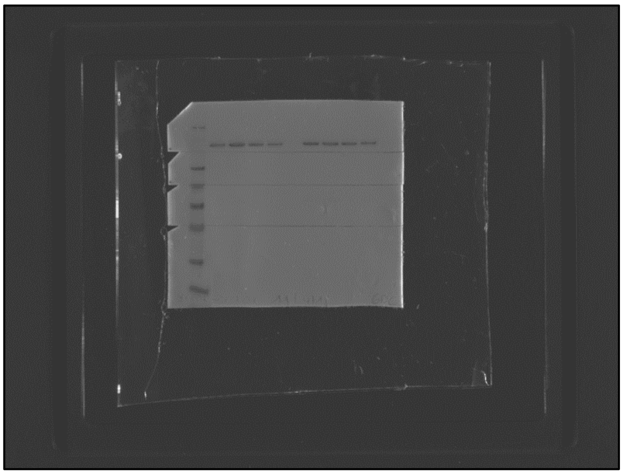

C

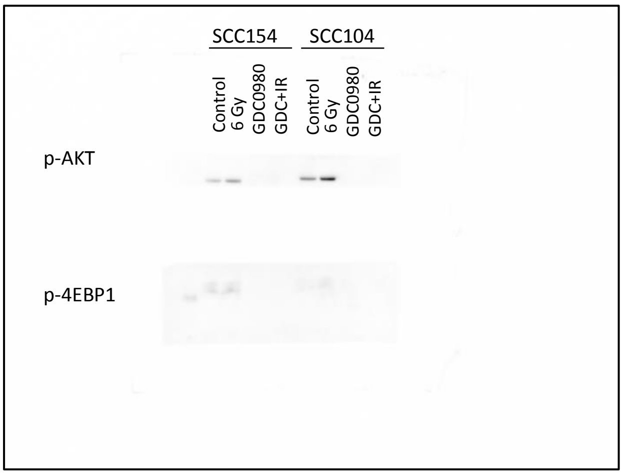

D

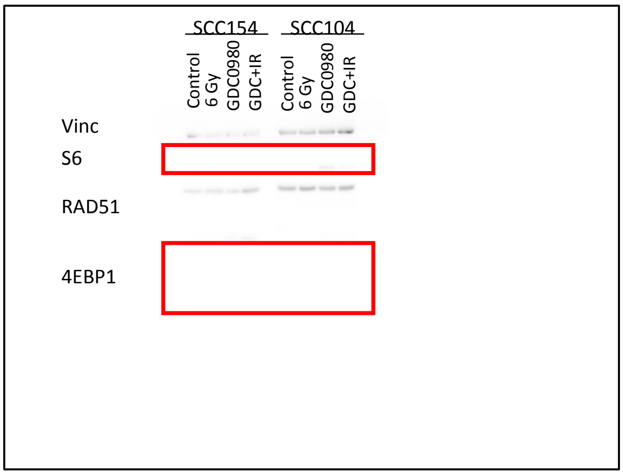

E

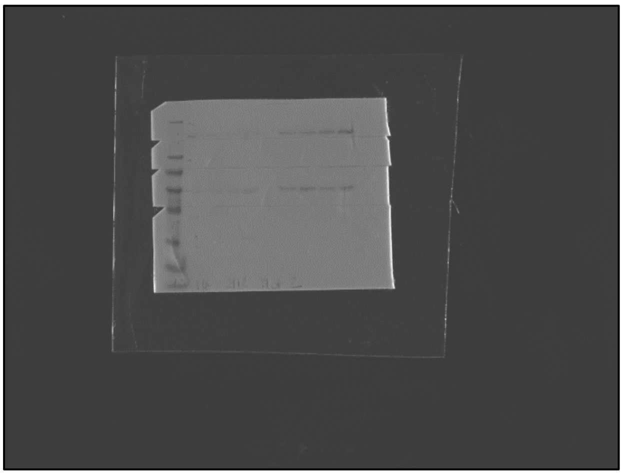

F

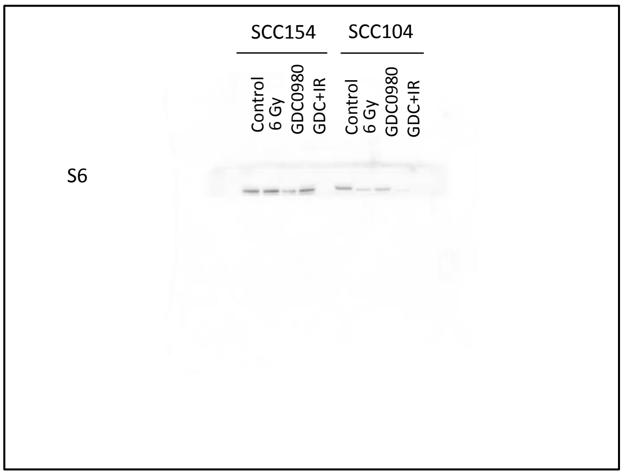
SCC154
SCC104
Control
6 Gy
GDC0980
GDC+IR
Control
6 Gy
GDC0980
GDC+IR
S6

G

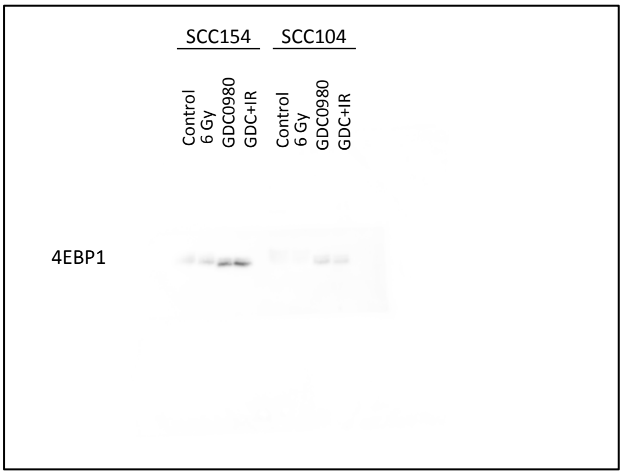
SCC154
SCC104
Control
6 Gy
GDC0980
GDC+IR
Control
6 Gy
GDC0980
GDC+IR
4EBP1

H

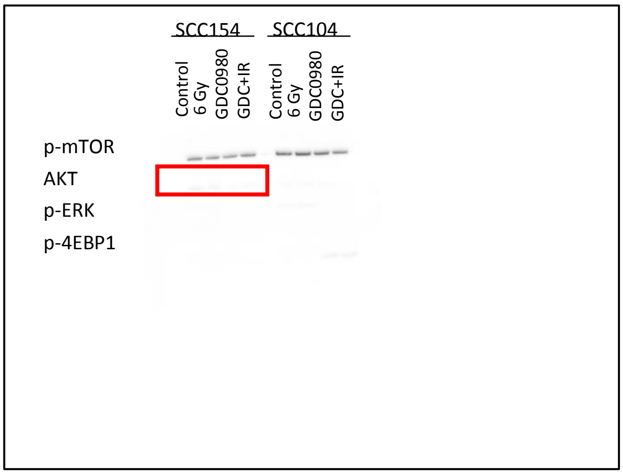
SCC154
SCC104
Control
6 Gy
GDC0980
GDC+IR
Control
6 Gy
GDC0980
GDC+IR
p-mTOR
AKT
p-ERK
p-4EBP1

I

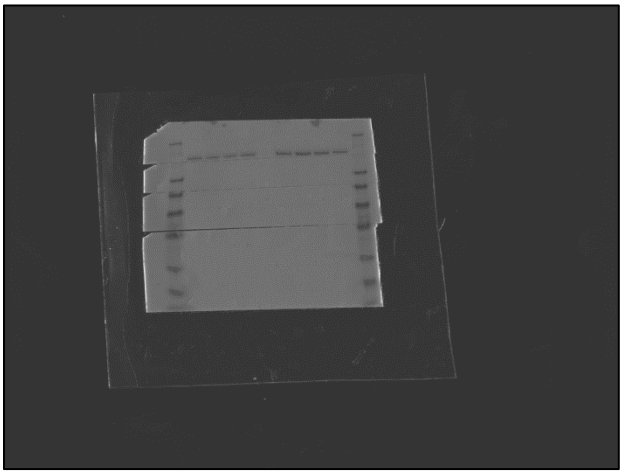

J

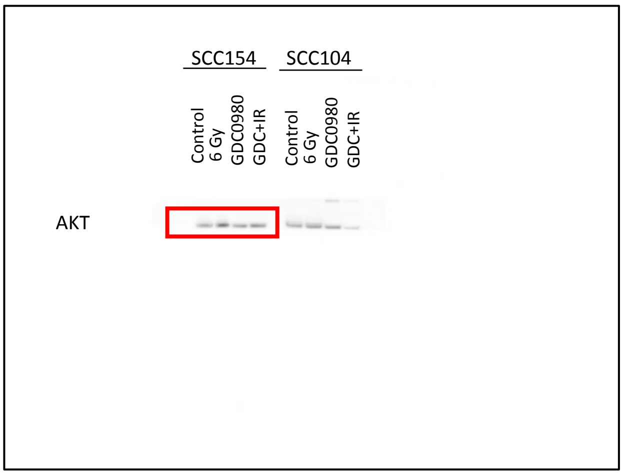
SCC154
SCC104
Control
6 Gy
GDC0980
GDC+IR
Control
6 Gy
GDC0980
GDC+IR
AKT

K

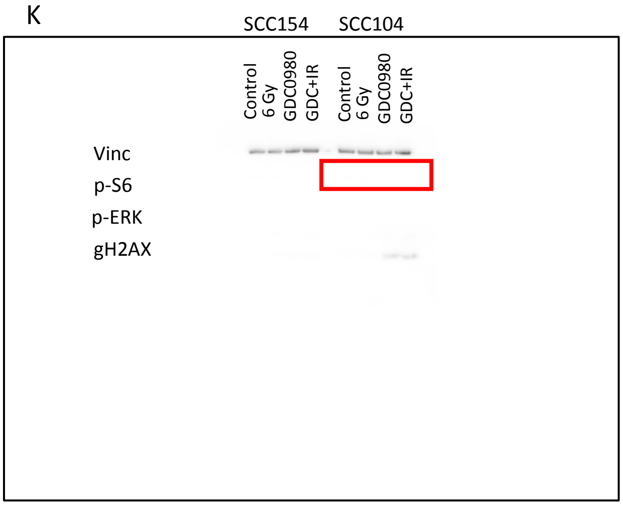
SCC154
SCC104
Control
6 Gy
GDC0980
GDC+IR
Control
6 Gy
GDC0980
GDC+IR
Vinc
p-S6
p-ERK
gH2AX

L

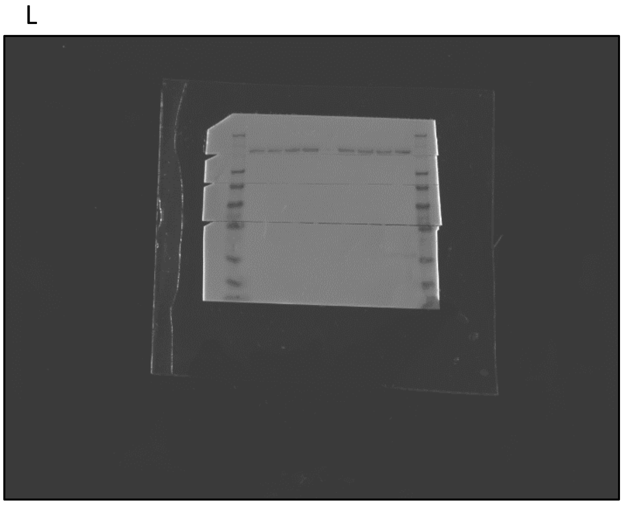

M

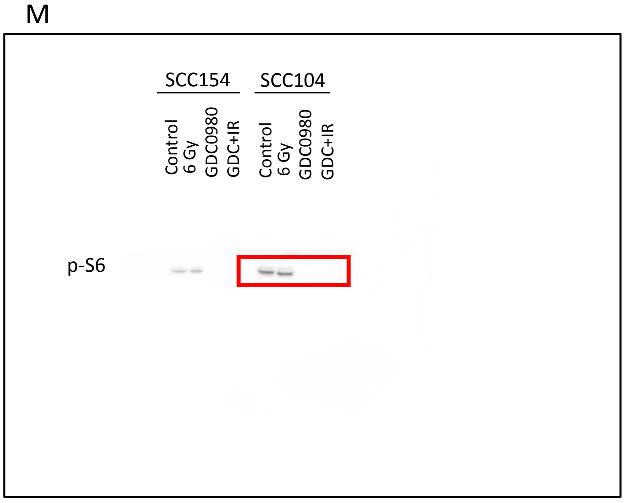
SCC154
SCC104
Control
6 Gy
GDC0980
GDC+IR
Control
6 Gy
GDC0980
GDC+IR
p-S6

N

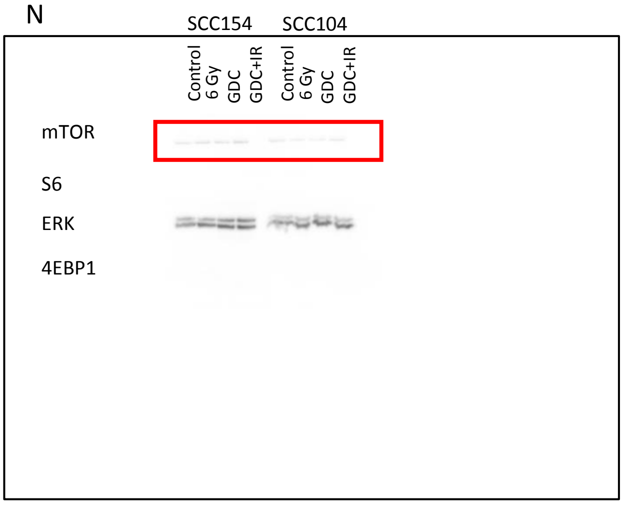
SCC154
SCC104
Control
6 Gy
GDC
GDC+IR
Control
6 Gy
GDC
GDC+IR
mTOR
S6
ERK
4EBP1

O

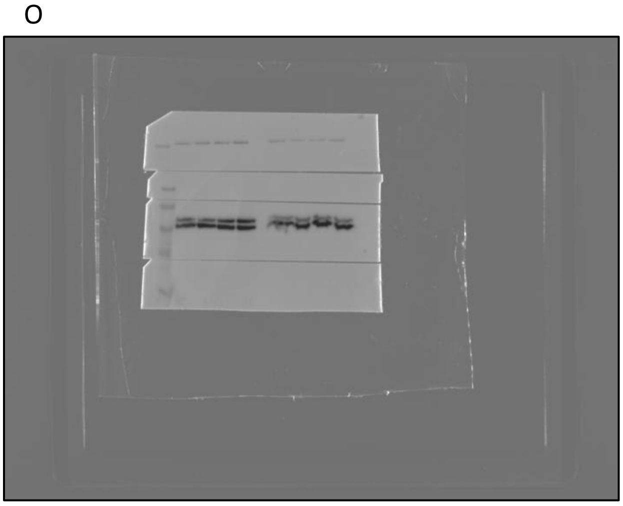

P

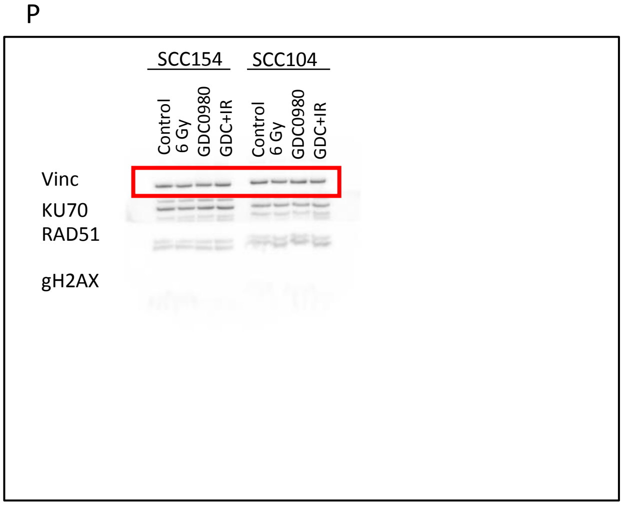
SCC154
SCC104
Control
6 Gy
GDC0980
GDC+IR
Control
6 Gy
GDC0980
GDC+IR
Vinc
KU70
RAD51
gH2AX

Q

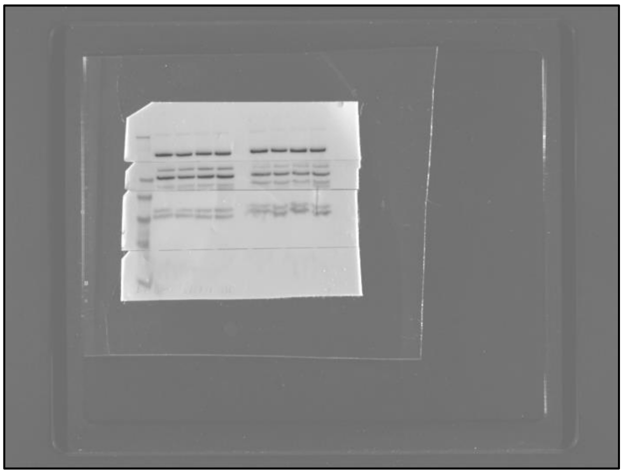

R

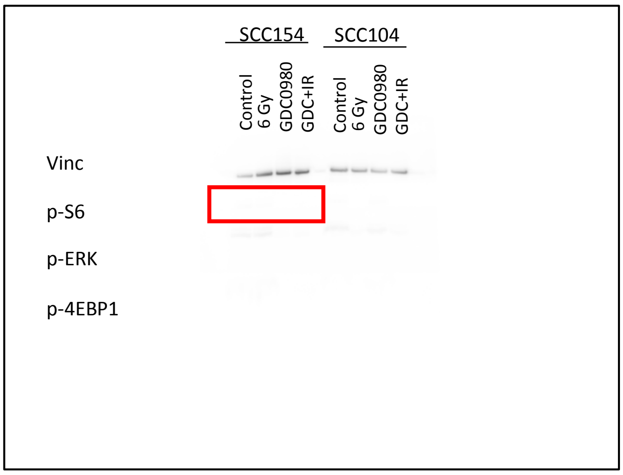
SCC154
SCC104
Control
6 Gy
GDC0980
GDC+IR
Control
6 Gy
GDC0980
GDC+IR
Vinc
p-S6
p-ERK
p-4EBP1

S

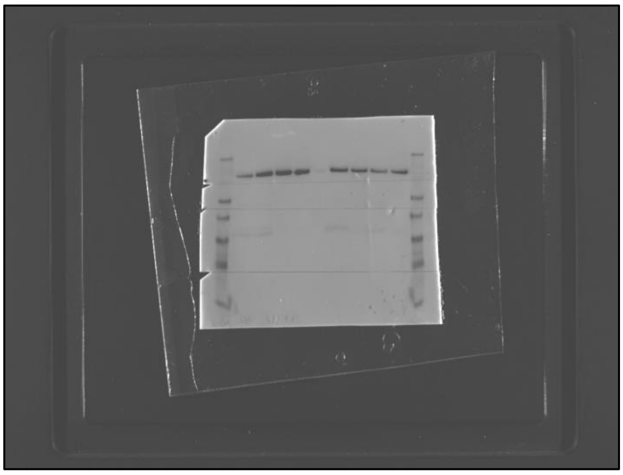

T

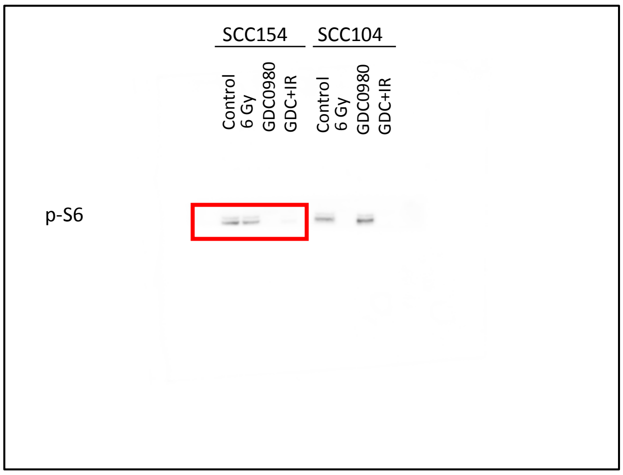
SCC154
SCC104
Control
6 Gy
GDC0980
GDC+IR
Control
6 Gy
GDC0980
GDC+IR
p-S6

U

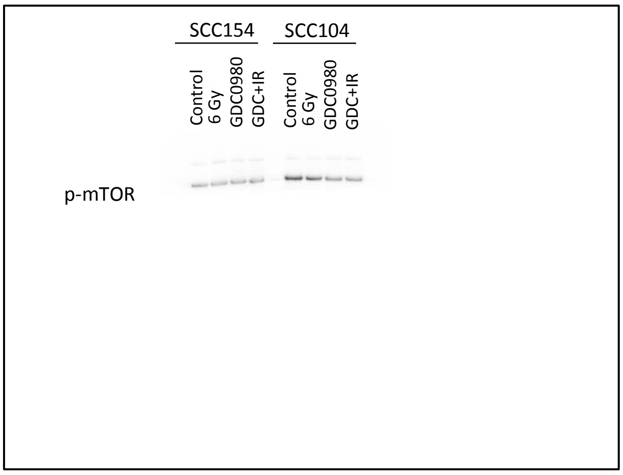
SCC154
SCC104
Control
6 Gy
GDC0980
GDC+IR
Control
6 Gy
GDC0980
GDC+IR
p-mTOR

V

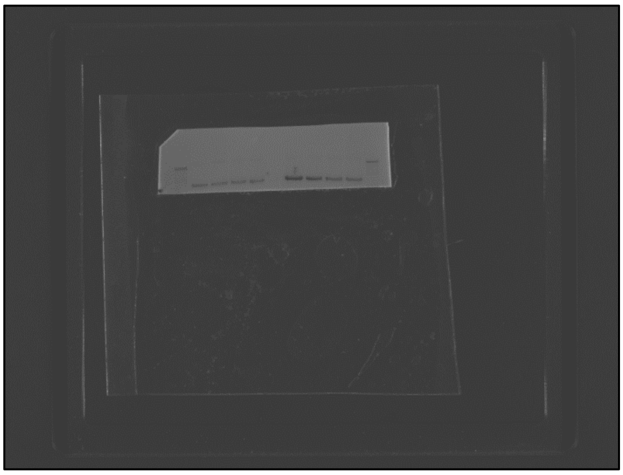

W

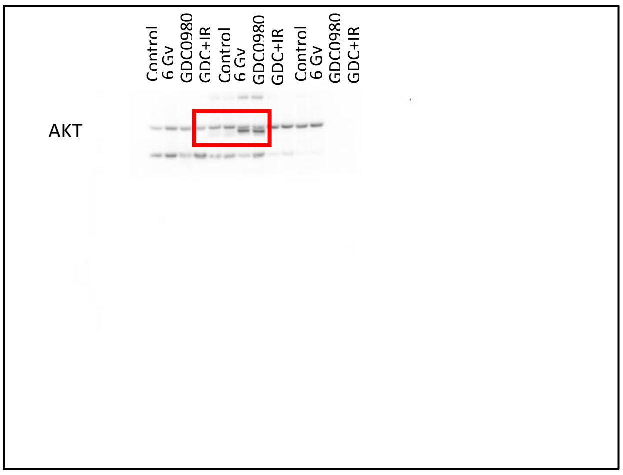

X

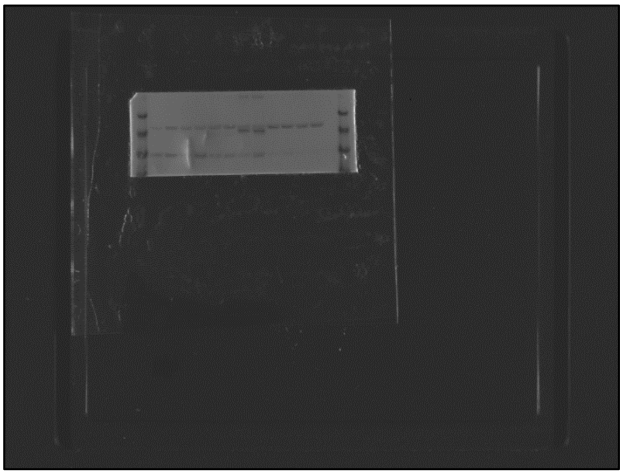

Supplementary Material 3. Uncropped western blot images of the head and neck cancer cell lines SCC154 and SCC104 used in Figure 3B. (A) Uncropped western blot of head and neck cancer cell lines SCC154 (lane 2-5) and SCC104 (lane 7-10) treated with vehicle, radiotherapy (6 Gy), GDC0980 (2.5 µM) or the combination of radiotherapy and GDC0980 (6 Gy and 2.5 µM) respectively (left to right). From top to bottom, the expression of vinculin, phospho-AKT, phospho-ERK and phospho-4EBP1 are visible in this blot, but only phospho-AKT, phospho-ERK and phospho-4EBP1 are used in Figure 3B (red box). **(B)** Overlay image with protein ladder of the uncropped western blot of Supplementary Figure 4A. **(C)** The second and bottom part of the blot shown in panel A and B had been developed separately to get a good visualization of the proteins phospho-AKT and phospho-4EBP1. **(D)** Uncropped western blot of head and neck cancer cell lines SCC154 (lane 2-5) and SCC104 (lane 7-10) treated with vehicle, radiotherapy (6 Gy), GDC0980 (2.5 µM) or the combination of radiotherapy and GDC0980 (6 Gy and 2.5 µM) respectively. The expression of vinculin, S6, RAD51 and 4EBP1 are visible in this blot, but only S6 and 4EBP1 are used in Figure 3B. **(E)** Overlay image with protein ladder of the uncropped western blot of Supplementary Figure 4 panel D. **(F)** The second part of the blot shown in panel D and E had been developed separately to get a good visualization of the protein S6. **(G)** The bottom part of the blot shown in panel D and E had been developed separately to get a good visualization of the protein 4EBP1. **(H)** Uncropped western blot of head and neck cancer cell lines SCC154 (lane 2-5) and SCC104 (lane 7-10) treated with vehicle, radiotherapy (6 Gy), GDC0980 (2.5 µM) or the combination of radiotherapy and GDC0980 (6 Gy and 2.5 µM) respectively. The expression of phospho-mTOR, AKT, phospho-ERK and phospho-4EBP1 are visible in this blot, but only AKT is used in Figure 3B. **(I)** Overlay image with protein ladder of the uncropped western blot of Supplementary Figure 4 panel H. **(J)** Second part of the blot shown in panel H and I had been developed separately to get a good visualization of the protein AKT of the cell line SCC154. **(K)** Uncropped western blot of head and neck cancer cell lines SCC154 (lane 2-5) and SCC104 (lane 7-10) treated with vehicle, radiotherapy (6 Gy), GDC0980 (2.5 µM) or the combination of radiotherapy and GDC0980 (6 Gy and 2.5µM) respectively. The same blot was used as in panel H. The expression of vinculin, phospho-S6, phospho-ERK and gH2AX are visible here, but only phospho-S6 is used in Figure 3B. **(L)** Overlay image with protein ladder of the uncropped western blot of Supplementary Figure 4 panel K. **(M)** Second part of the blot shown in panel K and L had been developed separately to get a good visualization of the protein

phospho-S6 of the ell line SCC104. **(N)** Uncropped western blot of head and neck cancer cell lines SCC154 (lane 2-5) and SCC104 (lane 7-10) treated with vehicle, radiotherapy (6 Gy), GDC0980 (2.5 µM) or the combination of radiotherapy and GDDC0980 (6 Gy and 2.5 µM) respectively. The expression of mTOR, S6, ERK and 4EBP1 are visible here, but only mTOR is used in Figure 3B. **(O)** Overlay image with protein ladder of the uncropped western blot of Supplementary Figure 4 panel N. **(P)** Uncropped western blot of head and neck cancer cell lines SCC154 (lane 2-5) and SCC104 (lane 7-10) treated with vehicle, radiotherapy (6 Gy), GDC0980 (2.5 µM) or the combination of radiotherapy and GDDC0980 (6 Gy and 2.5 µM) respectively. The same blot was used as in panel N. The expression of vinculin, Ku70, RAD51 and gH2AX are visible here, but only vinculin is used in Figure 3B. **(Q)** Overlay image with protein ladder of the uncropped western blot of Supplementary Figure 4 panel P. **(R)** Uncropped western blot of head and neck cancer cell lines SCC154 (lane 2-5) and SCC104 (lane 7-10) treated with vehicle, radiotherapy (6 Gy), GDC0980 (2.5 µM) or the combination of radiotherapy and GDDC0980 (6 Gy and 2.5 µM) respectively. The expression of vinculin, phospho-S6, phospho-ERK and phospho-4EBP1 are visible here, but only phospho-S6 of SCC154 is used in Figure 3B. **(S)** Overlay image with protein ladder of the uncropped western blot of Supplementary Figure 4 panel R. **(T)** Second part of the blot shown in panel R and S had been developed separately to get a good visualization of the protein phospho-S6. **(U)** Uncropped western blot of head and neck cancer cell lines SCC154 (lane 2-5) and SCC104 (lane 7-10) treated with vehicle, radiotherapy (6 Gy), GDC0980 (2.5 µM) or the combination of radiotherapy and GDDC0980 (6 Gy and 2.5 µM) respectively. The expression of phospho-mTOR is visible here, used in Figure 3B. The same blot was used as in Supplementary Figure 4 panel R and S. **(V)** Overlay image with protein ladder of the uncropped western blot of Supplementary Figure 4 panel U. **(W)** Uncropped western blot of head and neck cancer cell lines SCC154 (lane 2-5), SCC104 (lane 6-9) and SQD9 (lane 10-13) treated with vehicle, radiotherapy (6 Gy), GDC0980 (2.5 µM) or the combination of radiotherapy and GDDC0980 (6 Gy and 2.5 µM) respectively. The expression of AKT of SCC104 is visible here, used in Figure 3B. **(X)** Overlay image with protein ladder of the uncropped western blot of Supplementary Figure 4 panel W. For the ladder (SeeBlue^{TM}, Thermofisher) was used for all western blots.

Supplementary Material 4

A

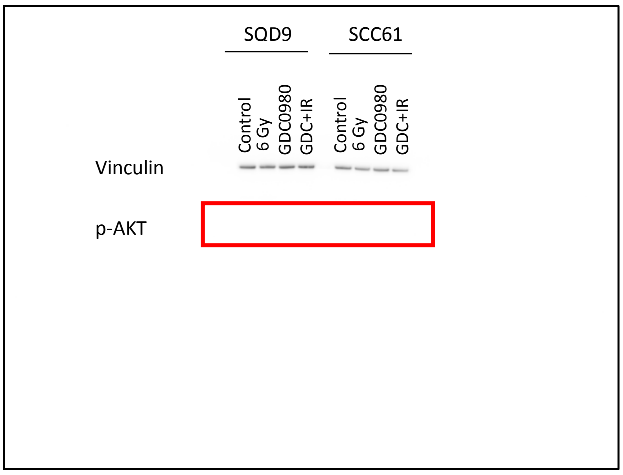

B

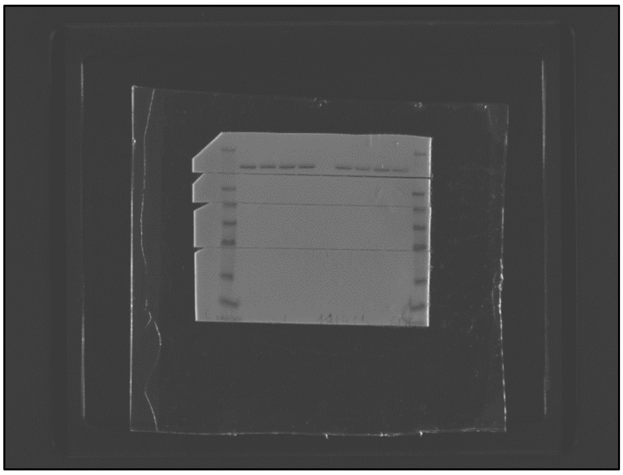

C

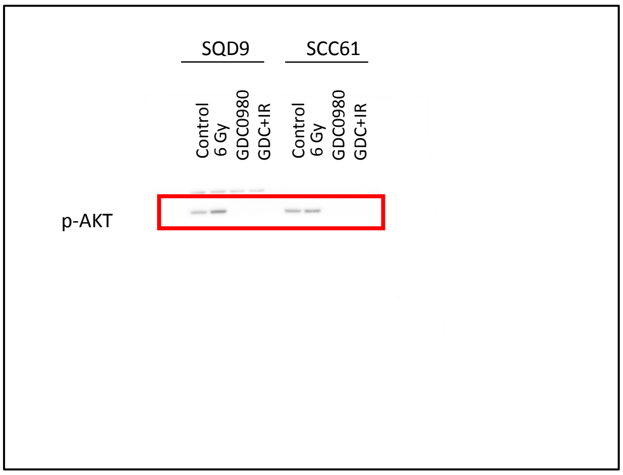
SQD9
SCC61
Control
6 Gy
GDC0980
GDC+IR
Control
6 Gy
GDC0980
GDC+IR
p-AKT

D

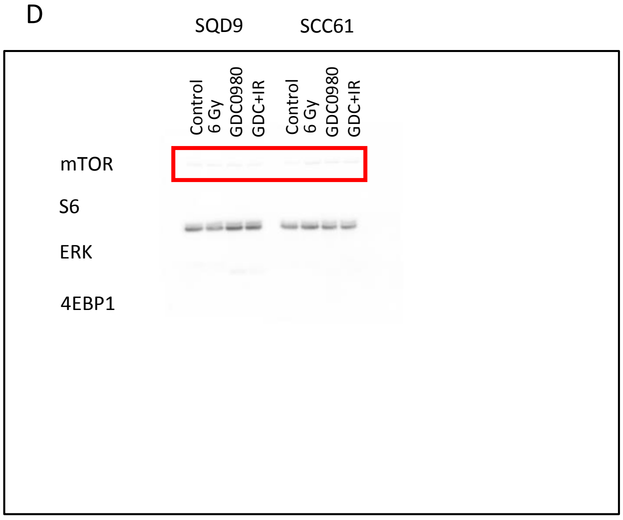
SQD9
SCC61
Control
6 Gy
GDC0980
GDC+IR
Control
6 Gy
GDC0980
GDC+IR
mTOR
S6
ERK
4EBP1

E

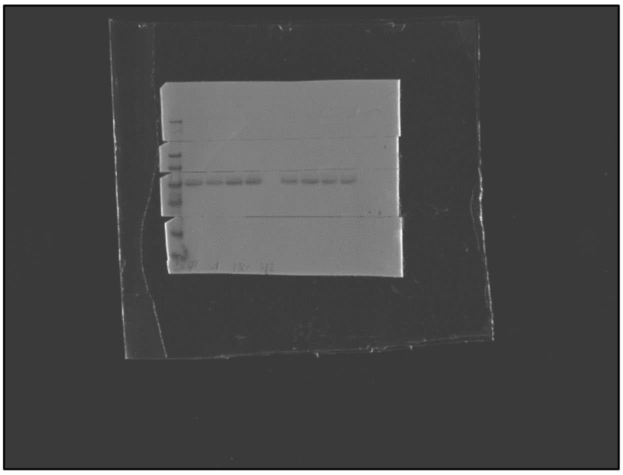

F

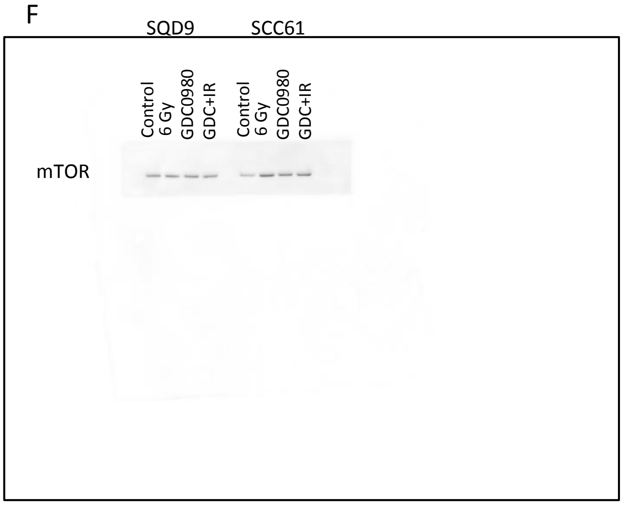
SQD9
SCC61
Control
6 Gy
GDC0980
GDC+IR
Control
6 Gy
GDC0980
GDC+IR
mTOR

G

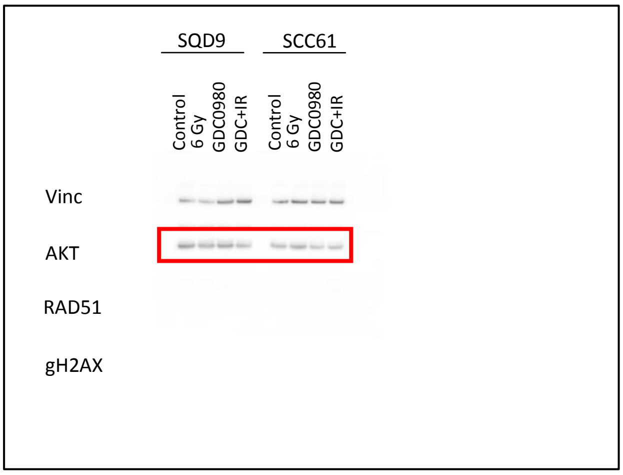
SQD9
SCC61
Control
6 Gy
GDC0980
GDC+IR
Control
6 Gy
GDC0980
GDC+IR
Vinc
AKT
RAD51
gH2AX

H

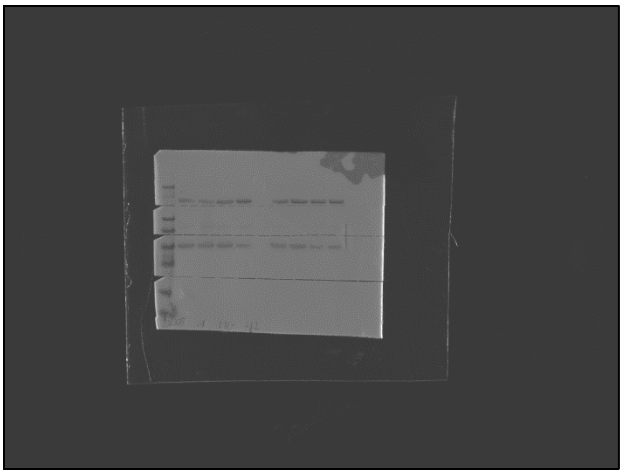

I

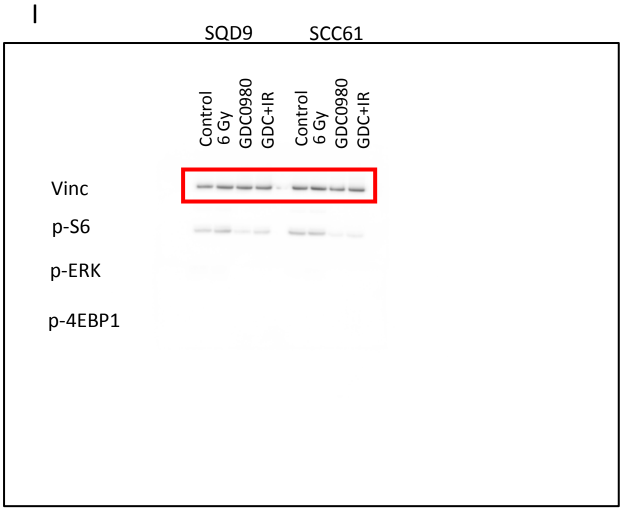
SQD9
SCC61
Control
6 Gy
GDC0980
GDC+IR
Control
6 Gy
GDC0980
GDC+IR
Vinc
p-S6
p-ERK
p-4EBP1

J

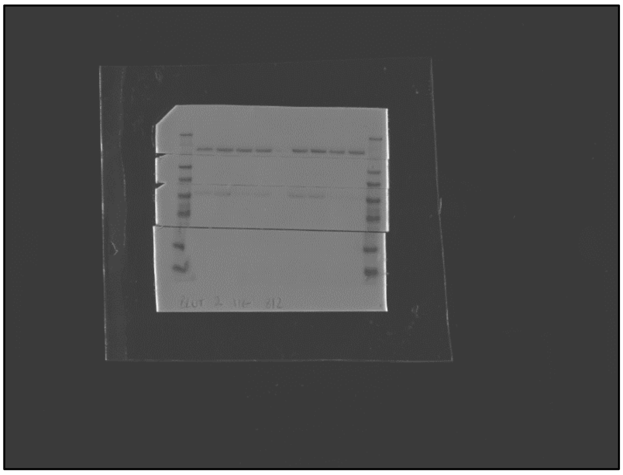

K

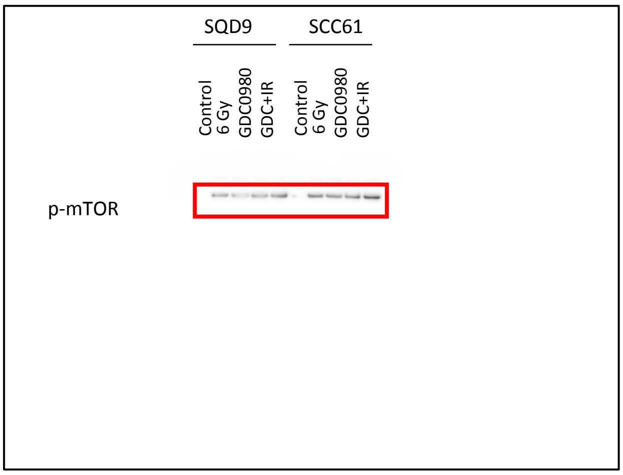
SQD9
SCC61
Control
6 Gy
GDC0980
GDC+IR
Control
6 Gy
GDC0980
GDC+IR
p-mTOR

L

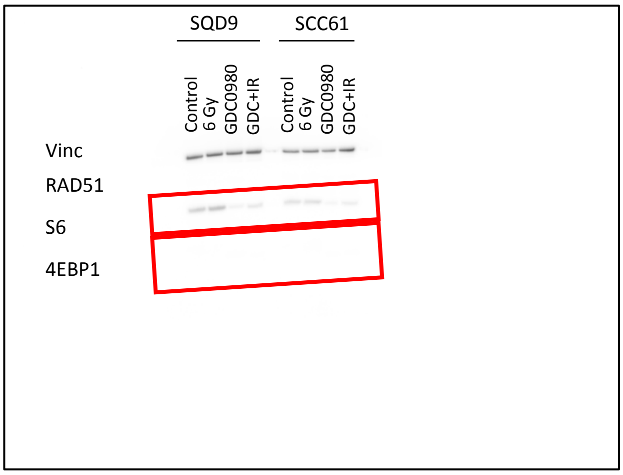
SQD9
SCC61
Control
6 Gy
GDC0980
GDC+IR
Control
6 Gy
GDC0980
GDC+IR
Vinc
RAD51
S6
4EBP1

M

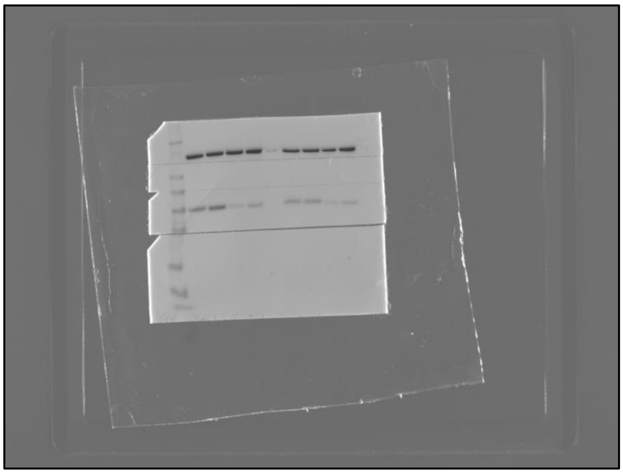

N

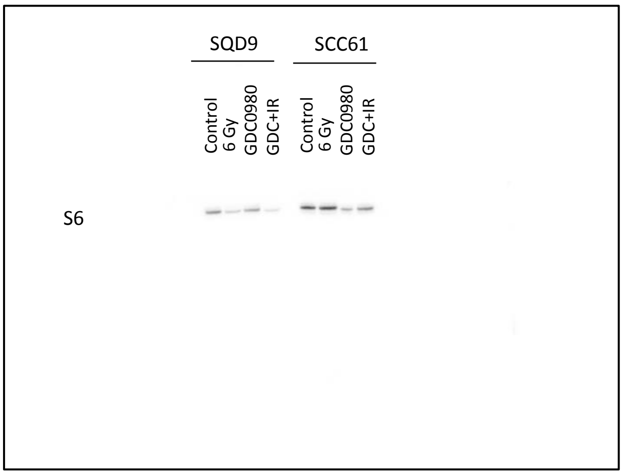
SQD9
SCC61
Control
6 Gy
GDC0980
GDC+IR
Control
6 Gy
GDC0980
GDC+IR
S6

O

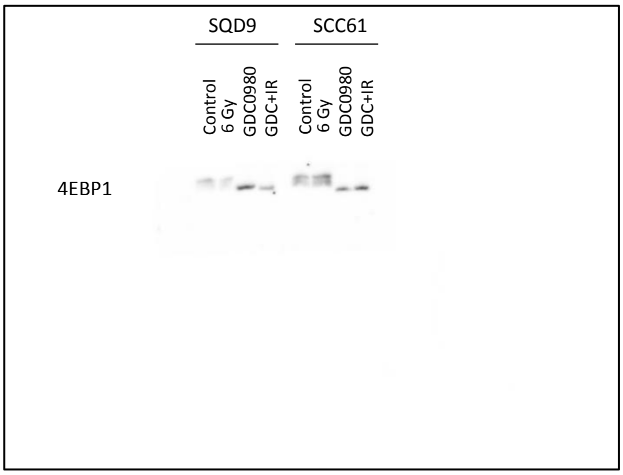
SQD9
SCC61
Control
6 Gy
GDC0980
GDC+IR
Control
6 Gy
GDC0980
GDC+IR
4EBP1

P

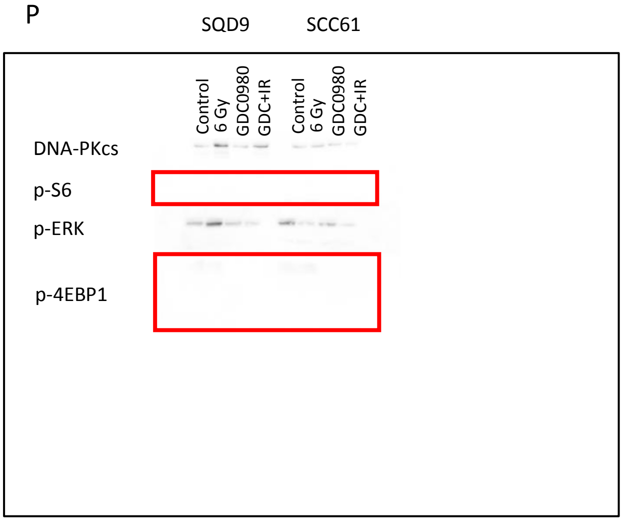
SQD9
SCC61
Control
6 Gy
GDC0980
GDC+IR
Control
6 Gy
GDC0980
GDC+IR
DNA-PKcs
p-S6
p-ERK
p-4EBP1

Q

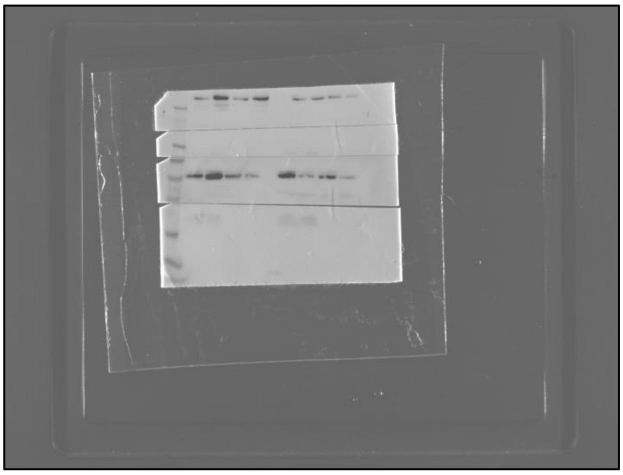

R

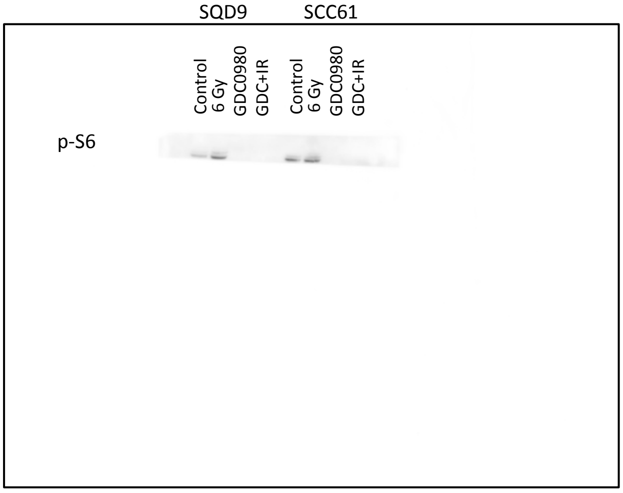
SQD9
SCC61
Control
6 Gy
GDC0980
GDC+IR
Control
6 Gy
GDC0980
GDC+IR
p-S6

S

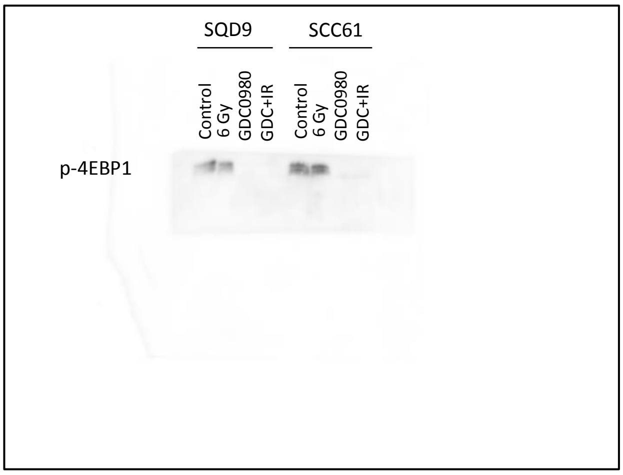
SQD9
SCC61
Control
6 Gy
GDC0980
GDC+IR
Control
6 Gy
GDC0980
GDC+IR
p-4EBP1

Supplementary Material 4. Uncropped western blot images of the head and neck cancer cell lines SQD9 and SCC61 used in Figure 3B. (A) Uncropped western blot of head and neck cancer cell lines SQD9 (lane 2-5) and SCC61 (lane 7-10) treated with vehicle, radiotherapy (6 Gy), GDC0980 (1 µM) or the combination of radiotherapy and GDC0980 (6 Gy and 1 µM) respectively (left to right). From top to bottom is the expression of vinculin and phospho-AKT visible on this blot, but only phospho-AKT is used in Figure 3B (red box). **(B)** Overlay image with protein ladder of the uncropped western blot of Supplementary Figure 5A. **(C)** The second part of the blot shown in panel A and B had been developed separately to get a good visualization of the protein phospho-AKT. **(D)** Uncropped western blot of head and neck cancer cell lines SQD9 (lane 2-5) and SCC61 (lane 7-10) treated with vehicle, radiotherapy (6 Gy), GDC0980 (1 µM) or the combination of radiotherapy and GDC0980 (6 Gy and 1 µM) respectively. The expression of mTOR, S6, ERK and 4EBP1 are visible on this blot, but only mTOR is used in Figure 3B. **(E)** Overlay image with protein ladder of the uncropped western blot of Supplementary Figure 5 panel D. **(F)** Same blot as panel D and E that had been developed separately to get a good visualization of the protein mTOR. **(G)** Uncropped western blot of head and neck cancer cell lines SQD9 (lane 2-5) and SCC61 (lane 7-10) treated with vehicle, radiotherapy (6 Gy), GDC0980 (1 µM) or the combination of radiotherapy and GDC0980 (6 Gy and 1 µM) respectively. The same blot was used as in panel D. The expression of vinculin, AKT, RAD51 and gH2AX are visible on this blot, but only AKT is used in Figure 3B. **(H)** Overlay image with protein ladder of the uncropped western blot of Supplementary Figure 5 panel G. **(I)** Uncropped western blot of head and neck cancer cell lines SQD9 (lane 2-5) and SCC61 (lane 7-10) treated with vehicle, radiotherapy (6 Gy), GDC0980 (1 µM) or the combination of radiotherapy and GDC0980 (6 Gy and 1 µM) respectively. The expression of vinculin, phospho-S6, phospho-ERK and phospho-4eBP1 are visible on this blot, but only vinculin is used in Figure 3B. **(J)** Overlay image with protein ladder of the uncropped western blot of Supplementary Figure 5 panel I. **(K)** Uncropped western blot of head and neck cancer cell lines SQD9 (lane 2-5) and SCC61 (lane 7-10) treated with vehicle, radiotherapy (6 Gy), GDC0980 (1 µM) or the combination of radiotherapy and GDC0980 (6 Gy and 1 µM) respectively. The same blot was used as in panel I. The expression of phospho-mTOR, is visible on this blot and used in Figure 3B. **(L)** Uncropped western blot of head and neck cancer cell lines SQD9 (lane 2-5) and SCC61 (lane 7-10) treated with vehicle, radiotherapy (6 Gy), GDC0980 (1 µM) or the combination of radiotherapy and GDC0980 (6 Gy and 1 µM) respectively. The expression of vinculin, S6, rad51 and 4EBP¨1 are visible on this blot, but only S6 and 4EBP1 are used in Figure 3B. **(M)** Overlay image with protein ladder of the uncropped western blot of Supplementary Figure 5 panel M. **(N)** Second part of the blot shown in panel M and N had been developed separately to get a good visualization of the protein S6. **(O)** Bottom part of the blot shown in panel M and N had been developed separately to get a good visualization of the protein 4EBP1. **(P)** Uncropped western blot of head and neck cancer cell lines SQD9 (lane 2-5) and SCC61 (lane 7-10) treated with vehicle, radiotherapy (6 Gy), GDC0980 (1 µM) or the combination of radiotherapy and GDC0980 (6 Gy and 1 µM) respectively. The expression of DNA-PK, phospho-S6, phospho-ERK and phospho-4EBP1 are visible on this blot, but only phospho-S6 and phospho-4EBP1 are used in Figure 3B. **(Q)** Overlay image with protein ladder of the uncropped western blot of Supplementary Figure 5 panel Q. **(R)** Second part of the blot shown in panel Q and R had been developed separately to get a good visualization of the protein phospho-S6. **(S)** Bottom part of the blot shown in panel M and N had been developed separately to get a good visualization of the protein phospho-4EBP1. For the protein standard, SeeBlue™(Thermofisher) was used for all western blots.
